# Repopulated microglia induce expression of *Cxcl13* with differential changes in Tau phosphorylation but do not impact amyloid pathology

**DOI:** 10.1186/s12974-022-02532-9

**Published:** 2022-07-04

**Authors:** Berke Karaahmet, Linh Le, Monique S. Mendes, Ania K. Majewska, M. Kerry O’Banion

**Affiliations:** 1 Department of Neuroscience, Del Monte Institute for Neuroscience, University of Rochester School of Medicine & Dentistry, Rochester, NY USA; 2 Department of Biology, Stanford University, Stanford, CA USA

**Keywords:** Alzheimer’s disease, Colony-stimulating factor 1, PLX5622, Microglia, RNAseq

## Abstract

**Background:**

Adult microglia rely on self-renewal through division to repopulate and sustain their numbers. However, with aging, microglia display morphological and transcriptional changes that reflect a heightened state of neuroinflammation. This state threatens aging neurons and other cells and can influence the progression of Alzheimer’s disease (AD). In this study, we sought to determine whether renewing microglia through a forced partial depletion/repopulation method could attenuate AD pathology in the 3xTg and APP/PS1 mouse models.

**Methods:**

We pharmacologically depleted the microglia of two cohorts of 21- to 22-month-old 3xTg mice and one cohort of 14-month-old APP/PS1 mice using PLX5622 formulated in chow for 2 weeks. Following depletion, we returned the mice to standard chow diet for 1 month to allow microglial repopulation. We assessed the effect of depletion and repopulation on AD pathology, microglial gene expression, and surface levels of homeostatic markers on microglia using immunohistochemistry, single-cell RNAseq and flow cytometry.

**Results:**

Although we did not identify a significant impact of microglial repopulation on amyloid pathology in either of the AD models, we observed differential changes in phosphorylated-Tau epitopes after repopulation in the 3xTg mice. We provide evidence that repopulated microglia in the hippocampal formation exhibited changes in the levels of homeostatic microglial markers. Lastly, we identified novel subpopulations of microglia by performing single-cell RNAseq analysis on CD45^int/+^ cells from hippocampi of control and repopulated 3xTg mice. In particular, one subpopulation induced after repopulation is characterized by heightened expression of *Cxcl13*.

**Conclusion:**

Overall, we found that depleting and repopulating microglia causes overexpression of microglial *Cxcl13* with disparate effects on Tau and amyloid pathologies.

**Supplementary Information:**

The online version contains supplementary material available at 10.1186/s12974-022-02532-9.

## Introduction

Microglia are the brain’s immune cells and are critical for normal brain function. In addition to their roles in maintaining tissue homeostasis, they are also key players in the pathogenesis and progression of Alzheimer’s disease (AD). Neuroinflammation associated with microglial changes is a cardinal feature of AD and leads to increased Tau pathology and hippocampal dysfunction [1–3]. Molecular mechanisms of the microglial response to AD pathology and the heterogeneity of microglial phenotypes in AD brains are the subject of intense debate. Several studies have identified a transcriptionally characterized cluster of microglia induced by aging and multiple neurodegenerative diseases, including AD, commonly referred to as disease-associated microglia (DAM) [4–8]. This cluster is defined by reduced expression of microglial homeostatic genes such as *Cx3cr1, P2ry12,* and *Tmem119* with concomitant upregulation of genes associated with lysosomal phagocytic activity and lipid metabolism such as *Apoe, Axl, Csf1, Clec7a, Cst7, Igf1, and Lpl* [4–8], some of which are implicated in AD pathogenesis [8–15]. Other clusters of microglia that accompany amyloid pathology have also been described, such as interferon response microglia (IRMs) that display upregulation of genes pertinent to interferon pathways [14, 16]. Although advances in single-cell RNA sequencing (scRNAseq) technologies have enabled the discovery of many novel clusters of microglia in homeostasis or disease, the full extent of microglial heterogeneity and its relationship to spatiotemporal properties of AD is still unclear.

To test whether microglia can alter AD pathogenesis, investigators have depleted microglia in the diseased brain using multiple techniques [17]. Recently, rapid partial depletion of microglia through oral administration of antagonists to colony-stimulating factor 1 receptor (CSF1R) signaling, which plays a role in microglial maintenance, has been widely utilized [18–21]. In the 5xFAD model of AD, inflammatory gene expression in the hippocampus was reversed with continuous depletion of microglia in aged mice, along with reduced neuritic damage and better performance on memory tests [19]. In aged 3xTg mice, sustained low-dose CSF1R inhibition of microglia showed an association between removal of microglia around amyloid-beta (Aβ) plaques and improved cognitive outcomes [18]. However, the effects of microglia elimination on amyloid plaque burden remain controversial, with data supporting no change [18, 19], exacerbation [22], or amelioration of amyloid pathology [20, 21]. Similarly, the impact of depletion on Tau pathology has been variable, perhaps due to the innate characteristics of the models used [23, 24]. Taken together, these findings reinforce the notion that microglia play active roles in AD, and depletion leads to variable changes during AD pathogenesis.

Adult microglia have a remarkable capacity to self-renew, and after depletion, repopulate their niche completely within 1 week, acquiring their normal densities, spacing, and morphological characteristics [25–29]. A number of studies have reported that depleting and repopulating microglia changes their phenotype to various degrees [30–34]. Here, we partially depleted microglia within the AD brain with PLX5622, a CSF1R inhibitor, and then allowed microglia to repopulate in order to investigate whether renewing microglia through this method could affect cognitive deficits, plaque formation, and Tau hyperphosphorylation in two mouse models of AD. While we observed changes in homeostatic markers in repopulated microglia, we did not see any differences in behavior between control and repopulated 3xTg mice. Microglial repopulation did not alter microglial phagocytosis of Aβ or amyloid plaque burden in either 3xTg or APP/PS1 mice. The impact of microglial renewal on Tau pathology was complex, with different Tau epitopes exhibiting different changes in phosphorylation. Interestingly, we found a novel enriched subpopulation of repopulated microglia characterized by upregulation of *Cxcl13.* CXCL13 is a small chemokine that can regulate lymphocyte homing and plays an important role in lymphoid neogenesis [35–37]. We visualized its expression in regions associated with AD pathology in PLX-treated 3xTg mice; however, the biological significance of *Cxcl13* requires further investigation. Overall, our data suggest that the repopulation of microglia after partial depletion induces a novel microglial phenotype that is associated with variable changes in Tau phosphorylation.

## Methods

### Experimental animals

All animal procedures were reviewed and approved by the University Committee on Animal Resources of the University of Rochester Medical Center and performed according to the Institutional Animal Care and Use Committee and guidelines from the National Institute of Health (NIH). Animals were housed in a 12-h light/12-h dark cycle with food ad libitum. 14-month-old male and female Tg(APPswe, PSEN1dE9)85Dbo mice (also known as APP/PS1) were obtained from an established colony (JAX stock no. 005864) maintained at the University of Rochester vivarium. Tg(APPSwe, tauP301L)1Lfa Psen1^tm1Mpm^ mice (also known as 3xTg) were initially obtained from Frank M. LaFerla and Salvatore Oddo by Howard Federoff and maintained at the University of Rochester as a homozygous line. The 3xTg mice express mutated human APP Swedish, MAPT P301L under the control of the Thy1.2 promoter and PS1 M146V under the Psen1 promoter. Age-matched non-transgenic (NTg) mice, bred continuously in a parallel colony to 3xTgs with a similar genetic background, were used as wild-type controls to 3xTg mice in RNA sequencing experiments. With age, the 3xTg mice develop Aβ plaque deposits and intraneuronal hyperphosphorylated Tau aggregates. Our studies used two cohorts of 21–22-month-old male 3xTg mice; females from these cohorts were not available as they had been used for unrelated studies.

### Microglia depletion and repopulation

Mice received a chow diet (AIN-76A-D1001i, Research Diets) containing 1200 mg/kg PLX5622 (Chemgood) ad libitum for 2 weeks to deplete microglia. Control chow with the same base formula without PLX5622 was given to the control group. After the 2-week treatment, mice were returned to the standard chow diet: AIN-76A (Research Diets) for the 3xTg Cohort 1 or 5053-Rodent Diet 20 (currently in use at the University of Rochester vivarium) to allow microglial repopulation for 1 month. All of these formulations were irradiated by the vendor.

### Behavioral assays

#### Open field (OF)

14 days before behavioral testing, mice were switched to a reverse light/dark cycle room. For 2 days before behavioral testing, mice were transported from the colony room to the behavior room, handled for ~ 5 min, and returned to the colony room on that day. On the day of testing, individual mice were placed in the center of a 31 × 31 cm box. After 20 s, the animal’s behavior was video recorded for 5 min. Mouse entries and time spent in the center zone and outside the zone were quantified using AnyMaze software (Stoelting Co).

#### Novel object recognition (NOR)

For the novel object recognition testing, mice were allowed to freely explore a 31 × 31 cm box containing two identical objects for 10 min. Doorknobs (5–6 cm in height and ~ 3 cm in width) were used. The testing chamber was sanitized between each trial with 70% ethanol. An hour after the habituation phase, mice were returned to the same box with one of the previously exposed, familiar objects and a novel object (i.e., a different doorknob). Placement of the novel object was randomized for each test. Mice were allowed to explore the box again for 5 min. Mice were videotaped during habituation and testing trials. For scoring, the time mice moved toward the object with the head facing the object and the neck extended was counted as exploratory behavior. Mice that spent less than 8 s exploring both objects were excluded from the analysis. A novel object discrimination index (DI) was defined by the following formula:

$$\text{Discrimination Index}= \frac{\text{Tn} - \text{Tf}}{\text{Tn} + \text{Tf}}$$

where Tf = time spent with familiar object and Tn = time spent with novel object.

#### Lashley maze

The Lashley III maze was used to test spatial memory in a stress-free environment [38]. The maze consisted of a start box, three interconnected alleys, and a pseudo-home cage. The alley was divided into zones. Mice were allowed to explore the maze for 10 min/day. The maze was sanitized with 70% ethanol between each trial and each animal. For 8 days, the number of zone entries was recorded under the assumption that mice that correctly remember the route from starting box to the pseudo-home cage will not enter zones outside of the direct path. Anymaze was used to record the videos.

#### Contextual fear conditioning (CFC)

After the behavioral tests above were conducted, mice underwent cued and contextual fear conditioning as previously described [39]. This paradigm was used to differentiate the contributions of the hippocampus which is required for learning of the context but not the cue associations [40]. Briefly, on the conditioning day, mice were allowed to explore the context that comprised an enclosed Plexi-glass chamber and a metal floor grid (model H10-11M, Coulbourn Instruments) inside an isolation chamber (Model H10-24T, Coulbourn Instruments). After 3 min, 15 s of white noise was presented, followed by a 2 s, 0.75 mA foot shock. The noise–shock pairing was repeated twice for a total of 3 shocks with 30 s intervals. The next day, mice were exposed to the same chamber for 5 min, and freezing behavior was quantified with AnyMaze. Four hours later, the mice were placed in a novel context (a plastic cylinder with bedding and red light) within the same Plexi-glass chamber. After 3 min, the conditioned tone stimulus was played, and freezing behavior was quantified.

### Flow cytometry/FACS

Mice were injected 24 h before euthanasia with Methoxy-X04 (MeX04, i.p., 4 mg/kg, Tocris Biosciences), a brain-permeable Aβ fluorescent marker [41, 42]. On the day of euthanasia, animals were deeply anesthetized with a mixture of xylazine (i.p., 10 mg/kg) and ketamine (i.p., 100 mg/kg) and perfused intracardially with 0.15 M phosphate buffer (PB) containing 0.5% sodium nitrite and 2 IU heparin/ml. After perfusion, hemispheres were separated: one was either immediately submerged in fixative solution (4% paraformaldehyde (PFA), pH 7.2 in PB, 4 °C) to be used for immunofluorescence experiments or flash-frozen in cold isopentane for ELISA (both as described below), and the other was processed for flow cytometry as follows. The hippocampus from each half brain was dissected and homogenized in 3 ml FACS buffer (1× Phosphate Buffered Saline (PBS) + 0.5% BSA). Homogenates were filtered through a 70 µm cell strainer into a 15 ml tube containing 3 ml FACS buffer. The strainer was washed with an additional 3 ml of FACS buffer, and the cell suspensions were centrifuged at 400×*g* for 5 min at 4 °C. The supernatants were discarded, and the remaining pellets were resuspended in 40% Percoll (Cytiva) prepared with PBS, then centrifuged at 400*g* for 30 min with no braking. After removing the supernatants, the pellets were resuspended in 90 µl FACS buffer with 1:100 Fc block (2.4G2, 1:100, BioLegend) and transferred to a 96-well plate. After a 15 min incubation with Fc block at 4 °C, the following antibodies were added in a 10 µl master mix: CD11b-FITC (M1/70, Biolegend), CD45-APC/Cy7 (30F11, Biolegend), 7AAD (Invitrogen), P2Ry12-APC (S16007D, Biolegend) & TMEM119-PE (106-6, Abcam). The latter two cell surface molecules are considered homeostatic microglial markers [4]. The plate was then incubated for 30 min at 4 °C in the dark. The samples were washed once with FACS buffer and transferred to 5 ml tubes containing 7AAD such that its final dilution was 1:80. Appropriate fluorescent-minus-one (FMO) and single-stained bead controls (Ultracomp eBeads, Invitrogen) were prepared in tandem with samples. After excluding debris, doublets, and dead cells, CD45^lo^/CD11b^+^ was used to gate for microglia on a FACSAria II (BD). MeX04^+^ and MeX04^−^ microglia were sorted. Samples from APP/PS1 mice were analyzed the same way, but with a LSR II flow cytometer (BD) without sorting. All events were recorded, and data were analyzed with FCS Express 7 (DeNovo Software).

### Immunofluorescence

Half-brains were fixed overnight in 4% PFA at 4 °C, dehydrated in 30% sucrose overnight, frozen in cold isopentane, and stored at − 80 °C until sectioning on a − 25 °C freezing stage microtome into 30 µm thick coronal slices stored in a cryoprotectant solution. For immunofluorescence, sections were washed extensively in PBS and blocked with 10% normal donkey or goat serum for 1 h at RT.

For amyloid pathology analysis, sections were immunolabeled for amyloid-beta (Aβ), microglia (Iba1 or P2RY12), a common microglial activation marker CD68, and a widely used marker for neuritic damage LAMP1 [43]. The following primary antibodies were used: biotin anti-Aβ (clone 6E10, 1:3000, BioLegend), rabbit anti-Iba1 (1:2000, Wako), rabbit anti-P2RY12 (1:2000, Anaspec), rat anti-CD68 (1:500, Bio-Rad) and rat anti-LAMP1 (1:2000, Abcam). Sections were incubated in primary antibodies for 48 h at 4 °C. The sections were washed and incubated in fluorescently labeled secondary antibodies/reagents (Alexa Fluor 488, Alexa Fluor 594 streptavidin conjugate and Alexa Fluor 647, Invitrogen; all at 1:1000) for 3 h at RT, then mounted and coverslipped (Prolong Gold, ThermoFisher Scientific).

For Tau pathology analysis, sections were incubated in biotinylated mouse anti-HT7 (1:1000, Invitrogen) and either rabbit anti-pT205 (1:1000, Invitrogen), rabbit anti-pS396 (1:1000, RayBiotech) or rabbit anti-pS409 (1:800, Invitrogen) for 2 h at RT then overnight at 4 °C. They were washed, incubated in fluorescently labeled secondary antibodies (Alexa Fluor 488, Invitrogen) and Alexa fluor 594 streptavidin conjugate (Invitrogen; all at 1:1000) for 3 h at RT, and then mounted and coverslipped.

### Image acquisition and analysis

For each animal, 3–4 coronal tissue sections that included the subiculum (S) and CA1 field of the hippocampus (CA1) were imaged with a Nikon A1R HD confocal microscope using a 10× (Plan Apo Lambda, NA: 0.40), 20× (Plan Apo VC, NA: 0.75) or 40× water-submersion (Apo LWD, NA: 1.15) objective lens as indicated in the figure legends. Imaging parameters were kept constant across all sections for each set of immunofluorescent labels. All image analysis was performed using ImageJ FIJI (NIH) with semi-automated custom macros. Experimenters were blinded to treatment.

#### Analysis of amyloid pathology and associated neuritic damage

For plaque area fraction, plaque size, and number analysis, regions of interest (ROIs) outlining the above-mentioned structures (S and CA1 for APP/PS1 and S for 3xTg) were drawn on maximum *z*-projections of the acquired 6E10 images. Images were subsequently thresholded and binarized using automated ImageJ’s Otsu thresholding algorithm, which was used for all other thresholding steps in this manuscript (except for MeX04 analysis, in which MaxEntropy was used). The plaque area fraction was calculated as the ratio between the number of pixels above the threshold over all pixels in the ROIs. The number of plaques per ROI was computed using automated ImageJ’s analyze particles function with a cut-off size of 50 µm^2^. Plaque size was calculated as the average size of all thresholded plaques above the 50 µm^2^ cut-off size.

For quantification of plaque-associated neuritic damage, 6E10 *z*-stacks were thresholded and binarized for analysis taking into account individual *z* planes. Subsequently, plaques detected by the analyze particles algorithm were dilated 25 µm to encompass the surrounding tissue for quantification of plaque-associated LAMP1. The overlap between LAMP1 and microglia was measured by multiplying the binarized LAMP1 and Iba1 (or P2RY12) image stacks. The resultant image was subtracted from the binarized LAMP1 image to obtain a non-microglial LAMP1 image, which was multiplied with the image containing dilated plaques to compute the overlap between LAMP1 and areas surrounding plaques. The number of colocalized signal pixels was calculated and divided by the number of thresholded 6E10 pixels (non-dilated) to obtain the ratio of LAMP1/plaque as quantification of plaque-associated dystrophic neurites.

#### Analysis of microglia

To measure the total volume occupied by microglia, Iba1 or P2RY12 *z*-stacks were thresholded and binarized using the same algorithm as described above. The percentage thresholded pixels was recorded as % microglia coverage. The 6E10 thresholded *z*-stacks mentioned above were dilated 5 µm, then plaque outlines were overlaid on Iba1 or P2RY12 *z*-stacks. The percentage of microglial area associated with plaque was calculated as the number of colocalized signal pixels divided by all microglial pixels.

To assess microglial activation, CD68 and P2RY12 markers were quantified. CD68 analysis was performed on APP/PS1 and the first cohort of 3xTg animals, while P2RY12 analysis was done on the second 3xTg cohort. CD68 *z*-stacks were thresholded and binarized within the same region. The overlap between CD68 and microglia was measured by multiplying the binarized CD68 and Iba1 *z*-stacks. The number of colocalized signal pixels was divided by the total microglia pixels to get the fraction of CD68-expressing microglia. For quantification of P2RY12 intensity as a proxy for microglia activation state, P2RY12 slices were summed in the *z*-direction and duplicated. One P2RY12 image was thresholded, and the P2RY12-negative region was chosen as the background ROI. On the other image, background intensity was measured on the P2RY12 *z*-sum projection within the pre-defined background ROI. The pixel value for background intensity was then subtracted from the entire image. Subsequently, P2RY12 intensity was measured.

#### Analysis of Tau pathology

For 3xTg animals that exhibit tauopathy, images containing CA1, the region with the highest accumulation of pathological Tau, were analyzed. The area fraction of total Tau (tTau, clone HT7) and the three phospho-Tau epitopes (pT205, pS396, pS409) were computed in a similar manner as described above for plaque analysis. The ratio between pT205, pS396 or pS409 and HT7 was calculated and reported.

### Single-cell RNA sequencing

#### Generation of microglia single-cell suspension for sequencing

3xTg control-chow treated, 3xTg PLX-repopulated, and non-transgenic control-chow treated (NTg) mice were perfused and processed as described above in “Flow cytometry”. All of the equipment was maintained at 4 °C, and the processing steps were done on ice. The only modifications were that the *F*_c_ block was incubated for 10 min, and primary antibodies were incubated for 20 min. The primary antibodies used were CD11b (M1/70) and CD45 (30F11) from BioLegend. DAPI (BD) was used as a viability stain. DAPI^−^CD45^int/+^ events were sorted on a BD FACSAria II using an 85-micron nozzle. Each sample took approximately 3–7 min to sort. Throughout the protocol, samples were kept on ice, and the FACSAria II was operated in a 4 °C environment. In our preliminary experiments, we identified over 85% viability with this method (data not shown). The samples were immediately processed for single-cell capture as described below.

#### Single-cell sequencing

Cellular suspensions containing 50,000–90,000 CD45^int/+^ events were loaded on a Chromium Single-Cell Instrument (10× Genomics, Pleasanton, CA, USA) to generate single-cell gel bead-in-emulsions (GEMs). Single-cell RNA-Seq libraries were prepared using Chromium Next GEM Single Cell 3′ GEM, Library & Gel Bead Kit v3.1 (10× Genomics). The beads were dissolved, and cells were lysed per the manufacturer’s recommendations. GEM reverse transcription (GEM-RT) was performed to produce a barcoded, full-length cDNA from poly-adenylated mRNA. After incubation, GEMs were broken, and the pooled post-GEM-RT reaction mixtures were recovered, and cDNA was purified with silane magnetic beads (DynaBeads MyOne Silane Beads, PN37002D, ThermoFisher Scientific). The entire purified post GEM-RT product was amplified by PCR. This amplification reaction generated sufficient material to construct a 3ʹ cDNA library. Enzymatic fragmentation and size selection was used to optimize the cDNA amplicon size, and indexed sequencing libraries were constructed by End Repair, A-tailing, Adaptor Ligation, and PCR. Final libraries contain the P5 and P7 priming sites used in Illumina bridge amplification. Sequence data were generated using Illumina’s NovaSeq 6000.

#### scRNAseq data analysis

CellRanger v3.1.0 pipeline was used to demultiplex, make fastq files and generate gene counts of expression data referenced to mm10-3.0.0. It was determined that pooled samples had 150,000–200,000 mean reads per cell. Over 95% of reads were mapped to the genome, and over 93% of reads were above the quality control score of Q30. The gene expression matrix was analyzed by Seurat v3.1.5 package. Genes detected in less than 3 cells, and ribosomal genes were excluded from the analysis. Cells expressing less than 200 unique genes/features, more than mean + 3 * standard deviation number of transcripts, or more than 5% mitochondrial genes were excluded from the analysis. Overall, this approach yielded 5943 cells for 3xTg control-chow group and 9885 cells for the 3xTg PLX-repopulated group. These two groups of cells were further compared for findings reported in the main figures. In Supplemental data, we also show a direct comparison of these two groups to the 4979 cells identified in the non-transgenic (NTg) group.

Following the above filtering criteria, the data were normalized, 2000 most variable features were selected, and their expression was scaled with the built-in functions of the Seurat package. The top 20 Principal Components (PCs) were used for subsequent clustering (resolution = 0.25) and UMAP dimension reduction. To directly compare 3xTg microglia with NTg microglia, we used the anchoring algorithm of the Seurat package since these mouse lineages are bred to homozygosity, and the mice are not littermates. No anchoring algorithm was used for comparisons between control and PLX-repopulated 3xTg microglia. The scMCA package was used for the initial annotation of the cells [44]. The clusters encompassing perivascular macrophages and microglia (PVMMicro) were manually annotated according to the list of differentially expressed features that were determined by the FindMarkers() function with default Wilcoxon rank-sum test and |logFC|> 0.25. Significantly (*p*adj < 0.05), up- and down-regulated features were used as input to ClusterProfiler v3.16.0 for overrepresentation analysis to identify significantly enriched gene sets. FindMarkers() function was also used to identify differentially expressed features between the two chow treatments.

### In situ hybridization

PFA-fixed brain slices were used for in situ hybridization. RNAScope multiplex V2 Assay (ACD Bio) was used to detect *Cxcl13* (ACD Bio, 406311) transcripts per manufacturer’s instructions with slight modifications. Specifically, tissue mounted on SuperFrost Plus slides (Fisher Scientific) was subjected to 5 min of antigen retrieval at ~ 100 °C and was digested for 30 min with Protease Plus (ACD Bio). Opal 520 dye (Akoya Biosciences) was used at 1:800 for the detection of transcripts. Negative and positive control probes and spleen tissue (data not shown) were stained in tandem with experimental samples.

### ELISA and Western blot

Frozen hippocampi were homogenized in Tissue Protein Extraction Reagent (ThermoFisher Scientific) at a concentration of 50 mg/ml with 1× Halt Protease and Phosphatase Inhibitor Single-Use Cocktail (ThermoFisher Scientific), vortexed and sonicated. The homogenates were centrifuged for 100,000×*g* for 1 h. The supernatant was collected as the soluble fraction; whereas the pellet was incubated in guanidinium-HCl pH 6.0 for 4 h and centrifuged at 100,000×*g* for 1 h. This new supernatant was collected as the insoluble fraction. For Aβ40 ELISAs (ThermoFisher Scientific), the soluble fraction was diluted at 1:20, and the insoluble fraction was diluted at 1:3000. For Aβ42 ELISAs (ThermoFisher Scientific), the soluble fraction was diluted 1:2, and the insoluble was diluted 1:30. The soluble fraction diluted at 1:3 was used as input to the CXCL13 ELISA kit (R&D Systems). All dilutions were established empirically.

### Statistical analysis

All statistical analyses were performed in Graphpad Prism v7.04. Comparisons between PLX and control-treated groups in male-only experiments were made using Student’s *t*-test. Comparisons of MeX04 and MFI of several homeostatic markers were made using two-way ANOVA with Bonferroni correction. All data points that represent individual animal averages are presented as mean ± SEM. *p < 0.05, **p < 0.01, ***p < 0.001.

## Results

### Pharmacologically induced microglial self-renewal does not ameliorate amyloid pathology or neuritic damage in 3xTg and APP/PS1 mouse models

**Fig. 1:**
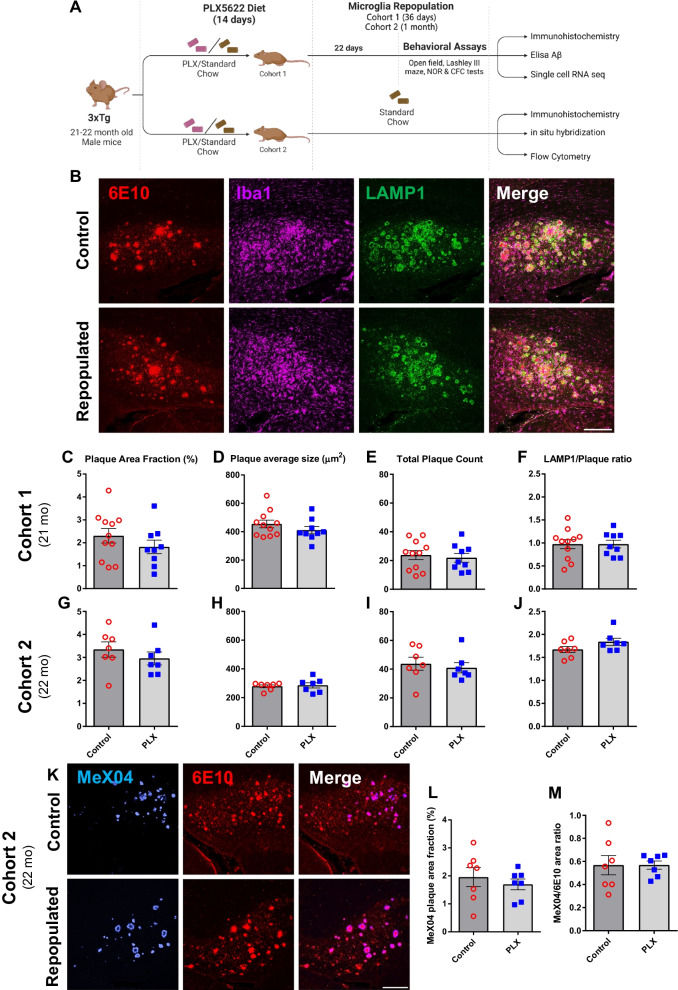
Repopulation of microglia does not ameliorate Aβ pathology and neuritic damage in male 3xTg mice. Experimental paradigm depicting duration of PLX5622 treatment and subsequent microglial repopulation (**A**). Representative confocal immunofluorescent 20× images of the subiculum in control versus PLX-repopulated group, showing Aβ plaque (6E10, red), microglia (Iba1, magenta), neuritic damage (LAMP1, green) (**B**). Scale bar represents 200 µm. There were no significant differences in the total area (**C**, **G**), size (**D**, **H**) and number (**E**, **I**) of plaques between the control and PLX-repopulated group in both cohorts. Ratio of plaque-associated LAMP1^+^ neuritic damage to plaque load was similar between the control and PLX-repopulated groups in both cohorts (**F**, **J**). Representative confocal immunofluorescent 20× images of the subiculum in control versus PLX-repopulated group, showing Aβ plaque labeled with MeX04 (blue) and 6E10 (red) (**K**). There was no difference in the total plaque area labeled with MeX04 (**L**) and the ratio of MeX04/6E10 (**M**) between the two treatment groups. Student’s *t*-test. Data are presented as mean ± SEM (Cohort 1: *n* = 9–11; Cohort 2: *n* = 7)

**Fig. 2:**
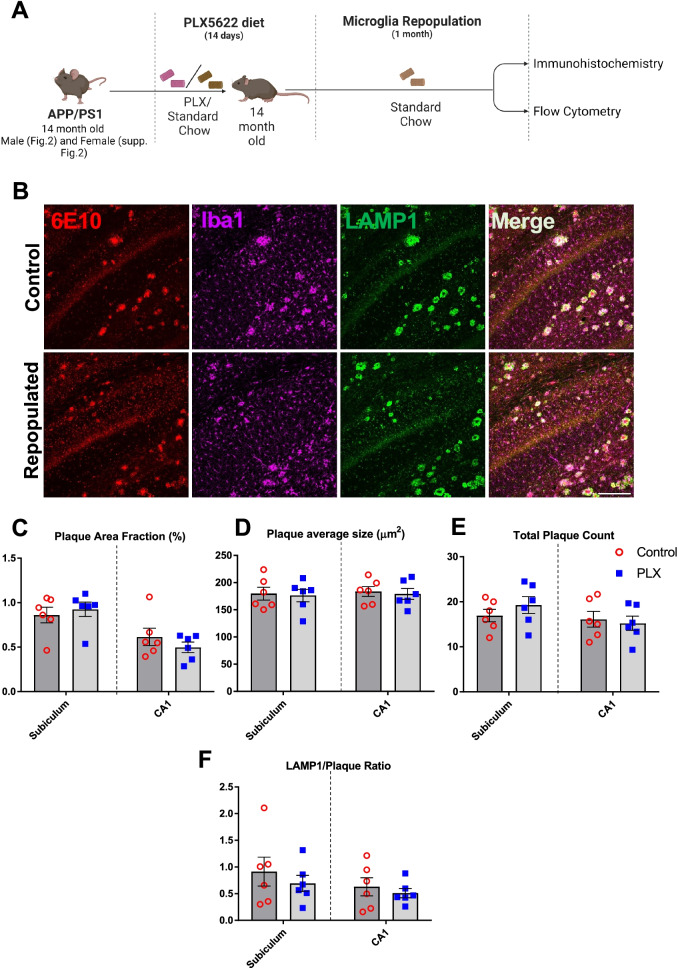
Repopulation of microglia does not ameliorate Aβ pathology and neuritic damage in APP/PS1 male mice. Experimental paradigm depicting duration of PLX5622 treatment and subsequent microglial repopulation (**A**). Representative immunofluorescent 20× images of the subiculum in control versus PLX-repopulated group, showing Aβ plaque (6E10, red), microglia (Iba1, magenta), and neuritic damage (LAMP1, green) (**B**). Scale bar represents 200 µm. There was no difference in the total area (**C**), size (**D**) and number (**E**) of plaques between the control and PLX-repopulated groups in Subiculum or CA1. Ratio of plaque-associated LAMP1^+^ neuritic damage to plaque load was similar between control and PLX-repopulated group (**F**). Student’s *t*-test. Data are presented as mean ± SEM (*n* = 6)

To explore the impact of microglial self-renewal on AD pathology, we utilized an established paradigm that partially depletes microglia using PLX5622 (PLX), a colony stimulating factor 1 receptor (CSF1R/c-kit/Flt3) inhibitor, in 3xTg and APP/PS1 mouse models of AD. After 2 weeks of PLX exposure, microglia numbers in the brains of aged 3xTg (22-month-old) and APP/PS1 (14-month-old) mice decreased by approximately 50% and 65%, respectively (Additional file 1: Fig. S1A–C, F). Consistent with previous findings [19], we observed greater depletion in plaque-devoid regions (50% in 3xTg and 70% in APP/PS1) versus 40% and 50% depletion of plaque-associated microglia in 3xTg and APP/PS1, respectively (Additional file 1: Fig. S1D, E, G, H). The impact of microglial repopulation was assessed 1 month after the discontinuation of PLX treatment (Figs. 1A, 2A). We found that self-renewal of microglia did not improve amyloid pathology, evidenced by the unaltered area fraction occupied by amyloid plaques, as well as the average size and number of plaques in either male 3xTg (Fig. 1B–E, G–I) or APP/PS1 mice of both sexes (Fig. 2B–E and Additional file 2: Fig. S2A–D).

Furthermore, when we examined dense-core plaques in 3xTg mice detected with MeX04, we observed no change with microglial repopulation (Fig. 1K–M). While ELISA measurements revealed a significant decrease of insoluble Aβ42, we did not detect any statistically significant changes in soluble forms of Aβ42 and Aβ40 or insoluble Aβ40 (Additional file 3: Fig. S3A–D). Since amyloid pathology only weakly correlates with neuronal loss and cognitive function [45], we sought to examine the impact of PLX treatment on the levels of dystrophic neurites in both APP/PS1 and 3xTg mice. In contrast to our expectations, we observed no improvement in levels of neuritic damage, indicated by similar levels of LAMP1 staining, a lysosomal membrane glycoprotein, under all conditions (Figs. 1F, J, 2F), except in the subiculum of female APP/PS1 mice, where repopulation increased LAMP1 levels (Additional file 2: Fig. S2E). Consistent with a lack of improvement in neuritic damage, microglial repopulation did not lead to changes in a battery of behavioral assays, including NOR, CFC, and Lashley III maze, in 3xTg mice (Additional file 4: Fig. S4A). Behavioral readouts for recognition, fear, spatial memory, and anxiety showed no difference in 21-month-old PLX-treated 3xTg mice when compared to control 3xTg mice (Additional file 4: Fig. S4B–F).

### Repopulated microglia exhibit no differences in their recruitment to and phagocytosis of amyloid plaques but show complex changes in the levels of activated and homeostatic markers

**Fig. 3:**
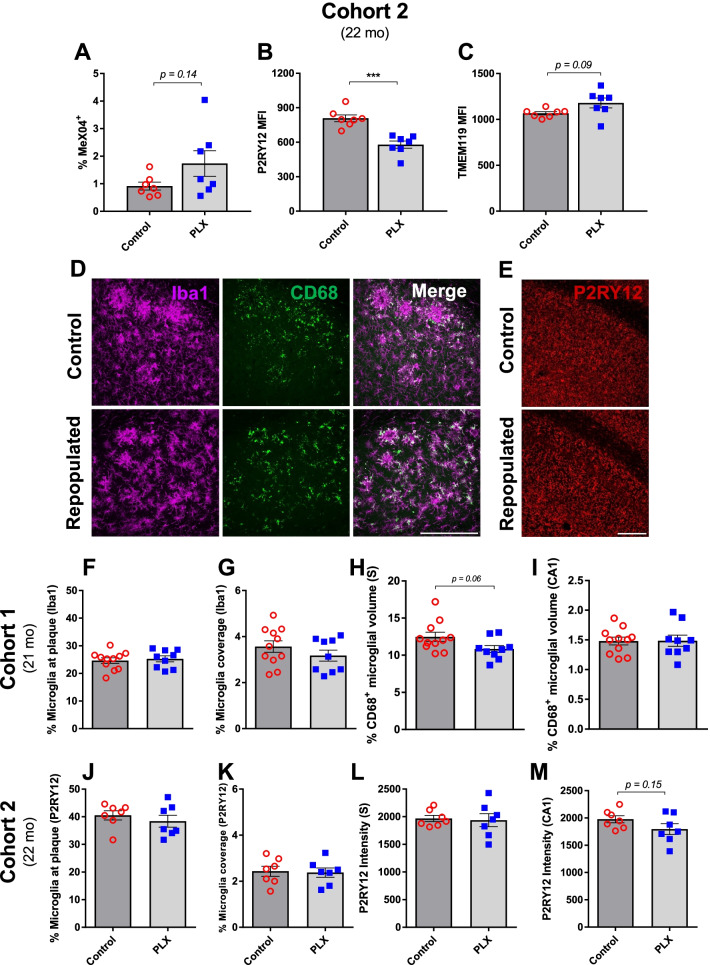
Repopulated microglia show similar recruitment to plaques but exhibit changes in homeostatic markers. Flow cytometry revealed no significant difference in microglia internalization of MeX04 in the two groups but showed a trend toward a higher percentage of MeX04^+^ microglia with PLX treatment (**A**). Microglial levels of P2RY12 (**B**) were significantly lower, while TMEM119 levels (**C**) were mildly elevated in PLX-repopulated group compared to the control. Representative immunofluorescent 40× images of the subiculum in the control and PLX-repopulated groups showing microglia (Iba1, magenta) and CD68 (green) in the first cohort of 3xTg mice (**D**). Representative immunofluorescent 20× images of the CA1 in the control and PLX-repopulated groups showing P2RY12 (red) in the second cohort of 3xTg mice (**E**). Scale bars represent 200 µm. Microglia in both cohorts showed no difference in their recruitment to plaque (**F**, **J**) as well as their total coverage (**G**, **K**) between control and PLX-repopulated groups. Levels of CD68, a marker of activated microglia, were slightly lower in the PLX-treated group in the subiculum (**H**) but not in the CA1 (**I**). The intensity of P2RY12, a homeostatic maker of microglia, was similar between treatments in the subiculum (**L**) but showed a trend toward being lower in the PLX-repopulated group in the CA1 region (**M**) as analyzed by immunohistochemistry. Student’s *t*-test (Welch’s corrections for **A** and **C**), ***p < *0.001*. Data are presented as mean ± SEM (Cohort 1: *n* = 9–11; Cohort 2: *n* = 7)

While microglial repopulation did not significantly attenuate plaque pathology or influence behavioral performance (Figs. 1 and 2; Additional file 2: Fig. S2, Additional file 3: Fig. S3, and Additional file 4: Fig. S4), we sought to determine whether repopulated microglia exhibited any changes in phagocytic potential or the levels of signature homeostatic and activation markers (Fig. 3). In order to assess microglial capacity to phagocytose plaques, we injected MeX04, a brain-permeable fluorescent probe for Aβ, 24 h prior to euthanasia and FACS-sorted hippocampal CD11b^+^CD45^int^ microglia into MeX04^+^ and MeX04^−^ fractions (Additional file 5: Fig. S5). The percentage of MeX04^+^ microglia, which represents the proportion of plaque-phagocytosing microglia, was not significantly different between PLX and control treatments, although there was a trend toward a higher fraction of microglia internalizing MeX04 with PLX treatment in the 3xTg model (3xTg: Fig. 3A, *p* = 0.14; APP/PS1: Additional file 6: Fig. S6A and Additional file 7: Fig. S7A). In agreement with these findings, microglia recruitment to plaque, quantified by the amount of Iba1 immunoreactivity in close proximity to plaque, also remained unaltered after repopulation (Fig. 3F, J; Additional file 6: Fig. S6E and Additional file 7: Fig. S7E). Similarly, we did not observe changes in total microglial volume coverage (Fig. 3G, K; Additional file 6: Fig. S6D and Additional file 7: Fig. S7D).

We next assessed the levels of CD68, P2RY12, and TMEM119 in repopulated microglia (Fig. 3B, C, H, I, L, M; Additional file 6: Fig. S6B, C, G and Additional file 7: Fig. S7B, C, F). Repopulated microglial levels of CD68, a well-known marker for reactive microglia, showed a trend toward decreasing in the subiculum of 3xTg mice (Fig. 3H, *p* = 0.06) and in the subiculum and CA1 in APP/PS1 mice, although the main effect of PLX treatment was only statistically significant in APP/PS1 female mice (Additional file 6: Fig. S6G and Additional file 7: Fig. S7F). Furthermore, microglial levels of CD68 were unchanged between the 3xTg groups in CA1, a region that does not display amyloid pathology in these mice (Fig. 3I). The levels of homeostatic markers P2RY12 and TMEM119 displayed more complex changes, reflecting the difference in pathologies between the two models. In the 3xTg model, the PLX treatment group displayed significantly lower levels of P2RY12 (Fig. 3B) but slightly higher levels of TMEM119 (Fig. 3C), albeit not significantly (*p* = 0.09). Due to the restricted amyloid pathology in the subiculum in 3xTg mice, the amount of MeX04^+^ microglia was too small to analyze separately and, therefore, was combined with MeX04^−^ microglia for analysis. Quantification of P2RY12 intensity in brain tissue revealed similarly decreased (although not significantly) levels of P2RY12 in the CA1 region of the hippocampus, a region with hyperphosphorylated Tau but lacking amyloid pathology (Fig. 3M, *p* = 0.15). This effect was not observed in the subiculum, which displays amyloid pathology (Fig. 3L). In the APP/PS1 model with only amyloid pathology, TMEM119 levels significantly increased with the repopulation of microglia in males but not in females (Additional file 6: Fig. S6B and Additional file 7: Fig. S7B). A similar pattern was observed with P2RY12 for males, which shows a trend (*p* = 0.15) toward higher levels with microglial repopulation (Additional file 6: Fig. S6C, *p*_F(treatment)_ < 0.05). As expected, MeX04^+^ microglia showed decreased levels of TMEM119 and P2RY12 compared to MeX04^−^ microglia in both sexes of APP/PS1 mice (Additional file 6: Fig. S6B, C and Additional file 7: Fig. S7B, C).

### Repopulated microglia impact phosphorylation of different Tau epitopes

**Fig. 4:**
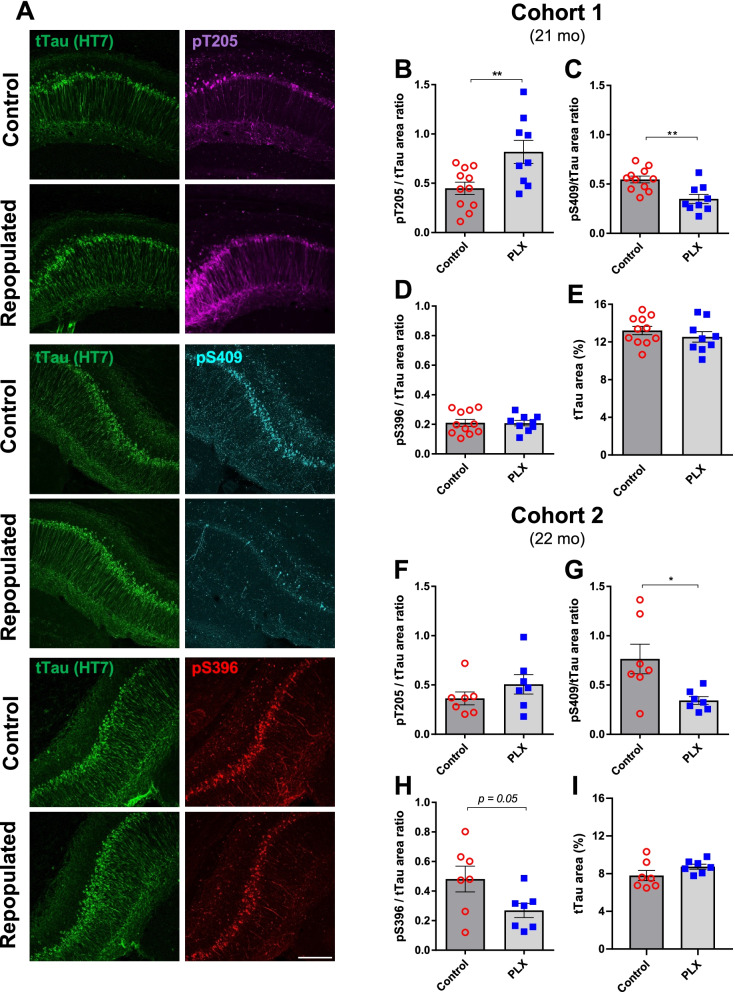
Repopulation of microglia impacts phosphorylation of different Tau epitopes in male 3xTg mice. Representative immunofluorescent 20× images of the CA1 hippocampal region in control and PLX-repopulated groups showing total Tau (HT7, green), pT205 (magenta), pS409 (cyan) and pS396 (red) (**A**). All images are from Cohort 1 and the scale bar represents 200 µm. Quantification of normalized pT205 revealed a significant increase in the PLX-repopulated group of Cohort 1 (**B**) but not Cohort 2 (**F**). On the other hand, normalized pS409 levels were found to be significantly decreased in Cohort 1 (**C**) and Cohort 2 (**G**). Levels of normalized pS396 were unchanged between control and PLX-repopulated groups of Cohort 1 (**D**), but showed a trend towards decreased levels in PLX-repopulated group of Cohort 2 (**H**). Levels of total Tau remained unchanged in both of the cohorts (**E**, **I**). Student’s *t*-test, *p < *0.05*. Data are presented as mean ± SEM (Cohort 1: *n* = 9–11; Cohort 2: *n* = 7)

To examine the effects of repopulated microglia on Tau pathology, we quantified the levels of three different phosphorylated Tau epitopes, pT205, pS409 and pS396, in two cohorts of 3xTg mice of similar age (Fig. 4A). When normalized to total Tau levels, we found that repopulation of microglia led to a significant increase in pT205 signal ratio in the first cohort (Fig. 4B) and a slight but non-significant increase in the second cohort (Fig. 4F). Interestingly, levels of pS409 normalized to total Tau were significantly decreased in both of the cohorts (Fig. 4C, G). Similarly, levels of pS396 showed a strong trend for a decrease (*p* = 0.05) in the second cohort but remained unchanged in the first cohort (Fig. 4D, H). Importantly, there was no change in levels of total Tau in any of the cohorts (Fig. 4E, I).

### scRNAseq reveals a microglia subset that expresses high levels of *Cxcl13* after repopulation in aged 3xTg mice

**Fig. 5:**
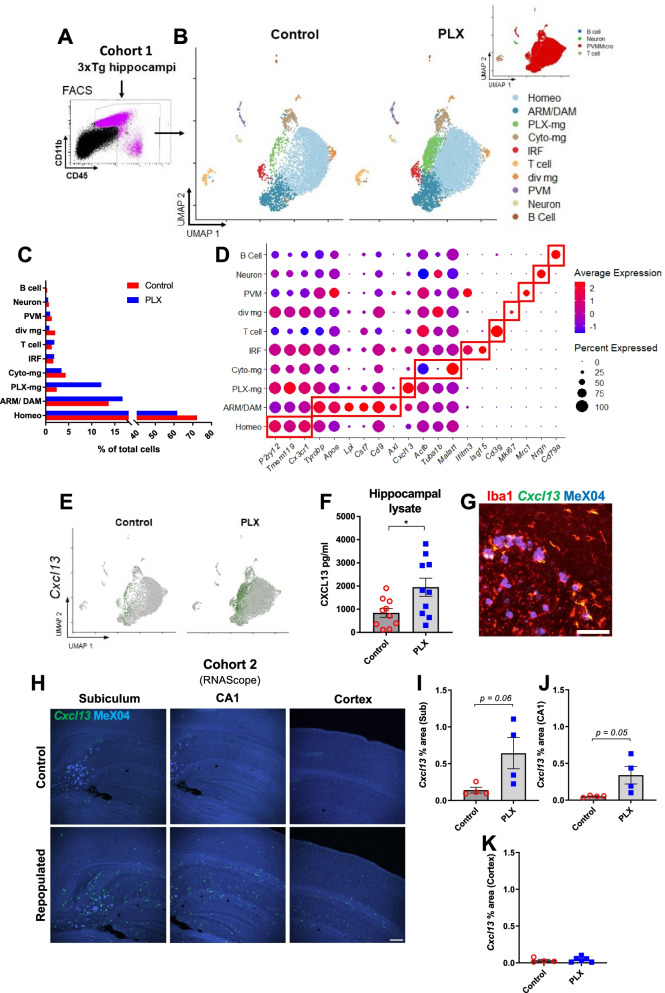
Repopulation increases *Cxcl13* expression in hippocampal microglia of male 3xTg mice. Schematic of experimental design (**A**, **B**). FACS-sorted cells were used as the input for scRNAseq (**A**). UMAP plots of control and PLX-repopulated groups illustrate clustering of cells (**B**). The inset shows the output of scMCA-based cell annotations. An overwhelming majority of sequenced cells were microglia (**B**). Proportions of different cell clusters out of all sequenced cells are depicted in **C**. Panel of genes identified through Seurat’s statistical framework or established literature is shown in **D** for annotation purposes. Red boxes highlight genes that are associated with their corresponding clusters. Increased *Cxcl13* expression can be noted across the majority of repopulated microglia but in particular for PLX-mg and ARM/DAM clusters (**E**). Increasing tones of green denote increased *Cxcl13* expression. ELISA on hippocampal lysates confirmed the upregulation of CXCL13 at the protein level (**F**). Representative immunofluorescent 10× images of in situ analysis of *Cxcl13* expression (**G**, **H**). Scale bar represents 100 µm in **G** or 200 µm in **H**. The resultant yellow signal from green *Cxcl13 *in situ hybridization and red Iba1 immunohistochemistry suggests that all of the *Cxcl13* transcripts we observed are associated with microglia (**G**). This representative image is from a PLX-repopulated sample. *Cxcl13* staining showed strong trends towards increased expression in PLX-repopulated groups in Subiculum and CA1 (**H**–**J**), however did not change in the cortex (**K**). Student’s *t*-test (**F**, **I**–**K**), *p < *0.05*. Data are presented as mean ± SEM

To determine transcriptomic associates of microglial repopulation, we performed single-cell RNA sequencing (scRNAseq) of FACS-isolated CD45^int/+^ cells from pooled hippocampi (Fig. 5A). After quality control, we identified 15,828 cells for further bioinformatic analysis. Cells identified as doublets or apoptotic were excluded as described in “Methods” section*.* Clustering analysis through the Seurat package revealed nine distinct clusters in our dataset, plotted on Uniform Manifold Approximation and Projection (UMAP) space, without any anchoring algorithms that may artificially coerce the position of individual data points (Fig. 5B). By using the single-cell Mouse Cell Atlas (scMCA package), we identified five major cell types: perivascular macrophages and microglia (PVMMicro), T cells, B cells, and neurons (Fig. 5B Inset). Microglial cells represented the overwhelming majority of identified cells and were further subdivided into six clusters (Fig. 5B–D).

In our dataset, most cells were identified as homeostatic microglia (Fig. 5C). These cells expressed markers such as *P2ry12*, *Cxc3cr1,* and *Tmem119* (Fig. 5D). The second most abundant cell type observed in both groups resembled activated response microglia (ARMs; or disease-associated microglia, DAM). This well-established cluster was identified based on the expression of *Tyrobp*, *Apoe*, *Lpl*, *Cst7,* and other markers in accordance with previous research (Fig. 5D) [3, 4, 14, 46]. Similarly, we identified a cluster enriched in interferon-related genes such as *Ifitm3* and *Isg15* (Fig. 5D). We believe these cells might be related to interferon response microglia (IRMs) which have also been previously observed [14]. We also observed a small cluster of microglial cells with high expression levels of cell cycle and DNA replication genes, such as *Mki67* (Fig. 5B, D), which could represent dividing microglia (“div mg”, also known as cycling/proliferating microglia or CPM). This small cluster has been identified in previous scRNAseq studies, too [14]. Together, these four clusters accounted for a similar proportion of total cells between control and repopulated microglia (Fig. 5C).

Furthermore, between 3 and 5% of CD45^int/+^ cells in the hippocampus fell into a microglial cluster defined by downregulation of cytoskeletal genes such as *Actb* (translates to β-actin) and *Tuba1b*; but also by upregulation of the long non-coding RNA *Malat1* (Fig. 5D). Gene Ontology analysis through clusterProfiler revealed that genesets pertinent to the regulation of actin, as well as mitochondrial function, were negatively enriched (Additional file 9: Fig. S9A, B). This cluster, which we refer to as “cyto-mg” for the purposes of this study, was found in similar proportions between control and PLX-repopulated (4.5% and 3.4%, respectively) groups but in much higher proportions in NTg mice (24.6%, Additional file 10: Fig. S10A–C).

Lastly, we identified a cluster (“PLX-microglia”) whose proportion changed between 3xTg control-treated and PLX-repopulated microglia (Fig. 5B). This “PLX-microglia” cluster made up 12.1% of CD45^int/+^ cells in the PLX-repopulated group but only 2.3% of cells in control-treated group in 3xTg mice (Fig. 5C). The PLX-microglia cluster was very strongly and almost exclusively (|logFC threshold|> 1.9, Additional file 11: Table S1), characterized by upregulation of *Cxcl13* (Fig. 5E). Detailed analysis of all microglial clusters revealed that PLX-repopulated microglia expressed more *Cxcl13* in general (Fig. 5E and Additional file 11: Table S1). In contrast, *Cxcl13* was detected in very few microglia isolated from control-treated NTg mice (Additional file 10: Fig. S10D). We confirmed increased CXCL13 levels in hippocampal lysates through ELISA (Fig. 5F), and we visualized *Cxcl13* mRNA expression through RNAScope technology (Fig. 5G, H). In our observations, *Cxcl13* staining colocalized with Iba1 immunopositivity, suggesting that *Cxcl13* is of microglial origin (Fig. 5G). We found strong trends towards increased *Cxcl13* expression in CA1 (*p* = 0.05) and subiculum (*p* = 0.06) in PLX-repopulated mice compared to the controls (Fig. 5I, J), but there was no meaningful difference between the groups in the cortex (*p* = 0.64, Fig. 5K).

## Discussion

In this present study, we investigated the impact of pharmacologically induced microglial renewal on pathology, microglial activation, and gene expression using both the 3xTg and APP/PS1 mouse models of Alzheimer’s disease. Previous work from our laboratory and others demonstrated that adult microglia are capable of rapid self-renewal following depletion [28, 47]. These new-born microglia are thought to be “rejuvenated” and beneficial in the context of disease settings such as toxin-induced acute neuronal lesion as well as normal aging [30, 31]. However, despite the emerging clinical relevance of targeting the neuroimmune system in AD patients [48], the long-term effects of microglial repopulation in the context of advanced-stage Alzheimer’s disease are only beginning to be explored [33]. Here, we demonstrated that 2 weeks of microglial depletion (which depleted more than 50% of the microglial population, Additional file 1: Fig. S1) followed by 1 month of microglial repopulation did not change amyloid pathology or levels of dystrophic neurites. However, we observed complex changes in Tau pathology and levels of specific markers of homeostasis on microglia. In addition, we identified a novel subpopulation of microglia enriched in the PLX-repopulated 3xTg microglia, which to the best of our knowledge, has not been described before.

The experimental intervention we used involves two separate manipulations, first depleting the microglia and then repopulating them. Therefore, one limitation of our design is that it cannot address the impact of a depletion-only paradigm which might have distinct, and perhaps opposite, effects on pathology, gene expression, and cellular activation compared to the repopulation phase. Although, we have considered depletion and repopulation as a single treatment for this article, future studies should dissect these to understand how short periods of microglial depletion affect AD pathology in the absence of repopulation.

### Repopulated microglia do not ameliorate amyloid pathology but exhibit differences in homeostatic markers in 3xTg and APP/PS1 mice

While microglia have complex roles in AD pathology, several studies have shown the benefits of eliminating microglia in AD mouse models. Although prolonged PLX treatment did not alter plaque load or Aβ levels in either 5xFAD (4-week PLX treatment starting at 14 months) [19] or 3xTg mice (12-week PLX treatment starting at 15-months) [18], both studies found that chronic microglial elimination partially prevented cognitive dysfunction in these mice [18, 19], suggesting that microglia contribute to neuronal dysfunction possibly via release of inflammatory cytokines and chemokines in the chronic neuroinflammatory environment [19]. Thus, “rejuvenating” microglia by pharmacologically inducing their repopulation could replace these microglia that have been shaped by their prolonged residence in an inflammatory environment, resulting in new microglia that better perform homeostatic functions. However, our experiments show that microglial repopulation did not ameliorate neuritic damage or improve cognitive outcomes in old AD-like animals (Figs. 1, 2 and Additional file 2: Fig. S2, Additional file 4: Fig. S4). Furthermore, we observed an increase in neuritic damage with microglial repopulation in APP/PS1 female mice, which is consistent with Gratuze et al*.* which described increased BACE1 immunopositivity around plaques in their depletion/repopulation paradigm, suggestive of increased dystrophic neurites [33]. We hypothesize that one potential explanation for our results could be that the repopulated microglia after PLX-depletion are “primed” by the surrounding inflammatory CNS microenvironment in the aged brain rapidly after they are born [32]. In fact, other investigators have shown that repopulated microglia do not alter the response to immune challenges or modify their expression of inflammation related genes [30, 32], which is consistent with our observations of the microglial activation marker CD68.

Interestingly, a recent study demonstrated that PLX treatment caused a significant shift from compact to diffuse plaque morphology with increasing neuritic damage [34]. These findings support a pivotal role for microglia in limiting fibrillar plaque expansion by encapsulating Aβ to form a protective barrier, preventing toxic effects of filamentous Aβ on nearby neurons [43, 49]. Disruption to microglial clustering around plaques led to a shift in plaque structure from compact to more diffuse with complex fibrillar branching, resulting in more dystrophic neurites surrounding plaques [43]. However, when microglia were allowed to repopulate, amyloid pathology was comparable to non-depleted animals, suggesting that the repopulated microglia replaced the resident population, but did not offer further disease-modifying benefits [34]. This is consistent with our observation that microglial repopulation did not alter amyloid pathology or improve dystrophic neurites surrounding plaques (Figs. 1, 2 and Additional file 2: Fig. S2). Although we observed a decrease in insoluble Aβ42 measured by ELISA, the insoluble Aβ40 and the soluble fractions of both Aβ42 and Aβ40 remained unchanged (Additional file 3: Fig. S3). Thus, our data add to previous findings and suggest no clear benefit of repopulation to amyloid pathology or cognitive outcomes (Figs. 1, 2 and Additional file 2: Fig. S2, Additional file 3: Fig. S3, and Additional file 4: Fig. S4) in 3xTg or APP/PS1 models of AD at stages of advanced pathology. In fact, repopulation may worsen pathology in some cases, as in a recent study which depleted and repopulated microglia in a Tau seeding model of 5-month-old 5xFAD mice and found that amyloid pathology was exacerbated 3.5 months post-PLX treatment [33]. On the other hand, chronic microglia depletion that is started before pathology onset (i.e., prior to plaque formation) led to a decrease in plaque burden and reduction of dystrophic neurites [20, 21], suggesting that intervening before the environment becomes inflammatory may lead to better outcomes. Simply limiting microglial proliferation beginning at an earlier timepoint in pathogenesis also reduces pathology [50], consistent with recent findings on the critical role of microglia in amyloid plaque seeding [9]. However, we believe microglial depletion after pathology onset is more relevant to clinical settings. Our data add to the growing body of evidence that repopulating microglia in advanced amyloid pathology does not offer clear benefits.

### Repopulated microglia impact phosphorylation of specific Tau epitopes

Since microglia play a complex role in the development of Tau pathology, the impact of their depletion and/or repopulation is of substantial clinical importance. The outcomes from depletion-only paradigms have revealed conflicting results depending on models used and dosages of CSF1R-antagonists administered [18–21, 51–53]. However, this is the first study to our best knowledge that has characterized the impact of microglial repopulation post-depletion on a transgenic model of Tau pathology. This is worth investigating since the side effects of sustained CSF1R inhibition can lead to uncharacterized outcomes [54]. We found that PLX-based repopulation of microglia increased pT205 immunopositivity, but not that of pS396 in CA1 (Fig. 4). A study in 5xFAD mice, in which Tau was seeded after the depletion and repopulation phases, also found increased AT8^+^ Tau immunoreactivity [33] which recognizes epitopes pS202 and pT205. On the other hand, we found that pS409 epitope was consistently and significantly decreased in the PLX-repopulated groups, while overall Tau levels were unchanged (Fig. 4). These findings highlight the value of assessing Tau pathology through multiple approaches [55] and suggest a potential disease-modifying benefit of our repopulation paradigm.

### Transcriptomic analysis of repopulated microglia identifies novel microglial subpopulations associated with PLX treatment and 3xTg phenotype

Transcriptomic analysis of microglial repopulation in 3xTg mice identified previously reported clusters of microglia associated with AD but also noted two distinct, previously unreported clusters. The “Cyto-mg” cluster was similarly represented in PLX-repopulated and control 3xTg mice, but its prevalence was greatly reduced compared to the NTg control mice (Additional file 10: Fig. S10A, B). Gene ontology analysis of downregulated transcriptomic markers that define this cluster revealed significant enrichment of gene sets pertinent to cytoskeleton organization and mitochondrion organization (Additional file 9: Fig. S9). The most upregulated gene in this cluster was *Malat1* (Fig. 5D, Additional file 11: Table S1), which has been shown to play roles in various neuroinflammatory insults—potentially through activation of the inflammasome [56]. However, we also acknowledge that downregulation of aforementioned gene ontology terms (Additional file 9: Fig. S9) may represent an artifactual outcome due to the lower number of detected RNA molecules or annotated genes (Additional file 8: Fig. S8). Further, it is unclear whether the transcriptomic signature of “Cyto-mg” cluster exists in microglia in other models of Tau pathology. Single-cell sequencing of microglia from Thy-Tau22 mice revealed a transcriptomic landscape characterized by increased proportions of ARM (or DAM) rather than a signature associated with cytoskeletal components or *Malat1* [57]. However, in that study, the authors anchor their Tau datasets with APP/PS1 datasets using CCA as a dimensionality reduction method (as opposed to PCA) which might have masked rare populations that exist only in one group [58]. Nevertheless, questions pertinent to existence and/or function of “Cyto-mg” cluster in models of AD are beyond the scope of this paper.

The other previously unreported cluster that we observed here is strongly defined by upregulation of *Cxcl13* (Fig. 5B, D). Although this “PLX-microglia” cluster was distinct from other types of activated microglia, we also observed *Cxcl13* upregulation in Homeo and ARM/DAM clusters, which account for most of the microglia, but not in other CD45^int/+^ cells (Fig. 5C, E and Additional file 11: Table S1). As expected, statistical testing on all cells identified as microglia revealed significant upregulation of *Cxcl13* in PLX-treated groups (Additional file 11: Table S1). We confirmed this finding at the protein level as well (Fig. 5F). Interestingly, we observed strong trends towards upregulation of *Cxcl13* transcripts with in situ hybridizations in CA1 and subiculum of PLX-repopulated mice, but this effect was not detected in the cortex (Fig. 5I–K). This suggests that *Cxcl13* is preferentially expressed in regions that display AD pathology. Since CXCL13 plays an important role in homing and activation of CXCR5^+^ lymphocytes, such as B cells, follicular helper T cells, and Th17 cells [37], this transcriptomic signature might suggest an overall increased inflammatory microenvironment. This is further supported by decreased microglial levels of P2RY12 (Fig. 3B). CXCL13 expression has also been observed in models of multiple sclerosis, particularly in cerebrospinal fluid and meningeal tertiary lymphoid organs [35]. Whether this leads to increased lymphocyte infiltration or formation of tertiary lymphoid organs following microglial repopulation in AD mouse models warrants further research.

Lastly, we found similar proportions of DAM/ARM between control and repopulated groups in 3xTg mice. This finding contrasts another study [33] in which microglia did not re-establish DAM signature following a repopulation paradigm in the 5xFAD model. Although it is difficult to directly compare across studies due to the use of different sequencing technologies that provide different levels of resolution (microarray vs scRNAseq), we argue that the age of animals (~ 5 months vs. 23 months), as well as the presence of active Tau pathology in our model, may have created an environment that is more conducive to re-establishment of the DAM/ARM signature.

## Concluding remarks

Taken altogether, we provide evidence that depleting and repopulating microglia in the context of ongoing simultaneous amyloid and tau pathologies can differentially impact hyperphosphorylation of specific isoforms of Tau despite having no apparent effect on Aβ plaque load. We did not observe a consistent effect of microglial repopulation on microglial reactivity but discovered that repopulation led to the emergence of a microglial subpopulation that strongly expresses *Cxcl13*. This signature was spatially associated with regions of pathology in the 3xTg mouse model.

## Supplementary Information

**Additional file 1: Figure S1.** 2 weeks of PLX5622 administration partially eliminates microglia in 3xTg and APP/PS1 mice. Representative confocal immunofluorescent 10× images showing plaques (6E10, red) and microglia (Iba1, magenta) in the cortex and hippocampus in control versus PLX groups in 3xTg (**A**) and APP/PS1 mice (**B**). Scale bar represents 200 µm. Quantification shows an approximately 50% and 65% decrease in total microglia coverage with PLX treatment in 3xTg and APP/PS1 mice, respectively (**C**, **F**). PLX depletes plaque-associated microglia by around 40% in 3xTg and 50% in APP/PS1 (**D**, **G**) as compared to non-plaque associated microglia which are depleted around 50% (3xTg, **E**) and 70% (APP/PS1, **H**). Student’s *t*-test, *p < 0.05, ***p < 0.001. Data are presented as mean ± SEM (*n* = 3–4).

**Additional file 2: Figure S2.** Repopulation of microglia does not ameliorate Aβ pathology and neuritic damage in APP/PS1 female mice. Representative immunofluorescent 20× images of the subiculum in control versus PLX-repopulated group, showing Aβ plaque (6E10, red), microglia (Iba1, magenta), and neuritic damage (LAMP1, green) (**A**). There was no difference in the total area (**B**), size (**C**) and number (**D**) of plaques between the control and PLX-treated groups. The ratio of plaque-associated neuritic damage to plaque load was similar between the control and PLX-repopulated group in CA1 but was significantly increased in the subiculum (**E**). Student’s *t*-test, *p < *0.05*. Data are presented as mean ± SEM (*n* = 6).

**Additional file 3: Figure S3.** Repopulation of microglia causes subtle changes in Aβ peptide levels. Hippocampal lysates from 3xTg Cohort 1 were processed to separate soluble and insoluble fractions, which were used as substrates for ELISA for Aβ42 (**A**, **C**) or Aβ40 (**B**, **D**). Student’s *t*-test, *p < *0.05*. Data are presented as mean ± SEM. *n* = 8–10.

**Additional file 4: Figure S4.** Repopulation of microglia does not alter behavior in 21-month-old male 3xTg mice. 21-month-old 3xTg mice were treated with control or PLX5622 chow for 14 days and then returned to control chow for the remainder of the experiment. After 22 days on control chow, behavioral assessments were conducted using Open Field, Novel Object Recognition, Lashley III maze, and Fear Conditioning (**A**). No differences were measured across groups in time spent in the open arena of the open field test (**B**) or the recognition index in the Novel Object Recognition task (**C**). To assess spatial memory in a stress-free environment the Lashley Maze was used as depicted. Mice were trained over 8 days to reach the pseudo-home cage from the start box in 10-min sessions each day. No change in the number of zone entries was observed between groups (**D**). No changes between the groups in the time spent freezing to the conditioned context (**E**) or the tone stimulus (**F**) were noted. Student’s *t*-test. Data are presented as mean ± SEM, *n* = 14–16.

**Additional file 5: Figure S5.** Gating strategy for 3xTg and APP/PS1 flow cytometry experiments. Briefly, debris was excluded based on SSC and FSC properties, and live cells were selected by gating for singlets and 7-AAD^−^ events. Microglia were defined as CD45^int^ CD11b^+^. MeX04 positive and negative events were determined with reference to APP/PS1, or non-transgenic C57BL/6 controls injected with MeX04 or vehicle (i.e., FMOs and negative controls). A representative sample from the second cohort of 3xTg mice is shown here. All flow cytometry experiments were gated as described above.

**Additional file 6: Figure S6.** Repopulated microglia in male APP/PS1 show similar recruitment to plaques but different homeostatic markers levels. Flow cytometry revealed similar internalization of MeX04 between control and PLX-treated groups (**A**). TMEM119 levels were higher in MeX04^−^ microglia in both conditions but was elevated by PLX treatment in both MeX04^+^ and MeX04^−^ microglia (**B**). While PLX treatment increased P2RY12 levels, the effect was most apparent in MeX04^+^ microglia, which had lower P2RY12 levels in the control group (**C**). There were no differences in total microglial coverage (**D**) as well as microglial recruitment to plaque (**E**) between control and PLX treatment. Representative immunofluorescent 20× images of the subiculum in control and PLX-treated groups showing microglia (Iba1, magenta) and CD68 (green) (**F**). Scale bar represents 200 µm. Microglial CD68 levels did not change in both subiculum and CA1 regions with PLX treatment (**G**). Student’s *t*-test *(A)* and Two-way ANOVA with Tukey’s post hoc tests *(B–E, G)*. Data are presented as mean ± SEM (*n* = 6).

**Additional file 7: Figure S7.** Repopulated microglia in female APP/PS1 show similar recruitment to plaques but different activated marker levels. Flow cytometry revealed similar internalization of MeX04 between control and PLX treatment groups (**A**). While both TMEM119 and P2RY12 levels were found to be lower in MeX04^+^ microglial, they did not change with PLX treatment (**B**, **C**). There was no difference in microglial total coverage (**D**) as well as their recruitment to plaque between treatments (**E**). Levels of microglial CD68 decreased in both subiculum and CA1 regions with PLX treatment (**F**). Student’s *t*-test *(A)* and Two-way ANOVA with Tukey’s post hoc tests *(B–F)*. Data are presented as mean ± SEM (*n* = 6).

**Additional file 8: Figure S8.** Quality Control metrics from scRNAseq dataset. Violin plots depicting the total number of detected RNA molecules (**A**), number of unique genes/features (**B**), and percentage of mitochondrial gene contamination (**C**) per cell post-quality control thresholding as described in “Methods” section.

**Additional file 9: Figure S9.** Gene Ontology Analysis of “Cyto-mg” cluster. Overrepresentation test (hypergeometric test) of genes detected to be significantly (*p*adj < 0.05) up- (**A**) or down-regulated (**B**) by Seurat’s FindMarkers() function performed through clusterProfiler package.

**Additional file 10: Figure S10.** Comparisons of AD control and repopulated microglial gene expression with a non-AD control. The same dataset as shown in Fig. 4 is illustrated in relation to NTg, control-treated mice on UMAP space (**A**). All experimental groups were prepared in the same way and on the same day within < 3 h. However, since the 3xTg and NTg lines were separately maintained as homozygous lines for over a decade, Seurat’s anchoring algorithm was used to compare the data. Bar plot showing the proportions of CD45^int/+^ cells sequenced per cluster (**B**). Dot plot showing the expression of several important genes used in annotating the clusters (**C**). Comparison of *Cxcl13* expression between control and PLX-treated 3xTg microglia and control-treated NTg microglia (**D**). Note the very few detected *Cxcl13* transcripts in NTg microglia compared to 3xTg groups.

**Additional file 11: Table S1.** Transcriptomic markers of clusters identified in Fig. 5.

## Data Availability

The raw data supporting the conclusions of this article will be made available by the authors, without undue reservation, to any qualified researcher upon request. The sequencing dataset supporting the conclusions of this article is available in Gene Expression Omnibus repository under Accession number GSE190607.

## Abbreviations

AD: Alzheimer’s disease
CNS: Central nervous system
OF: Open field
CFC: Contextual fear conditioning
NOR: Novel object recognition
Aβ: Amyloid beta
ELISA: Enzyme-linked immunosorbent assay
MeX04: Methoxy‑04
CSF1R: Colony-stimulating factor 1 receptor
pS396: Phosphorylated tau at serine 396
pS409: Phosphorylated tau and serine 409
pT206: Phosphorylated tau at threonine 206
scRNAseq: Single-cell RNA sequencing
DAM: Disease associated microglia
ARM: Activated response microglia
IRM: Interferon response microglia
*Cxcl13*: C-X-C motif chemokine ligand 13
P2RY12: Purinergic receptor P2Y12
TMEM119: Transmembrane protein 119
