# Supplementary figures and images for: Repopulated microglia induce expression of Cxcl13 with differential changes in Tau phosphorylation but do not impact amyloid pathology
Source: J Neuroinflammation. 2022 Jul 4;19:173. doi: 10.1186/s12974-022-02532-9 (PMC9252071; doi:10.1186/s12974-022-02532-9)

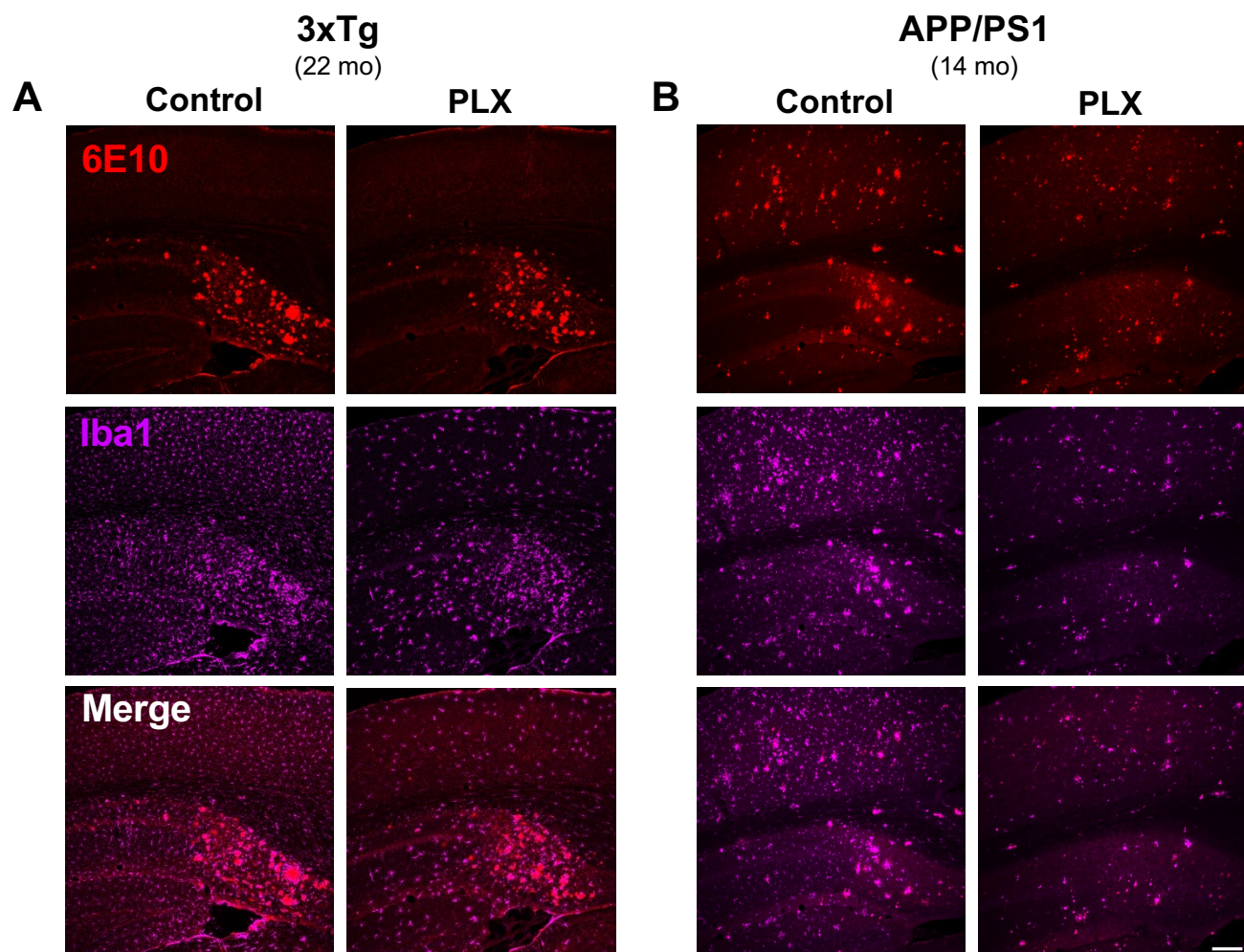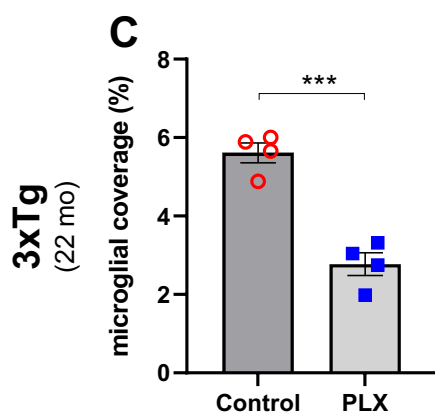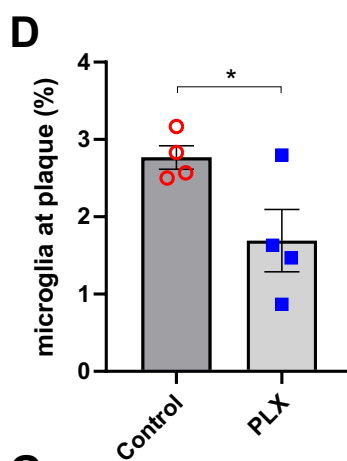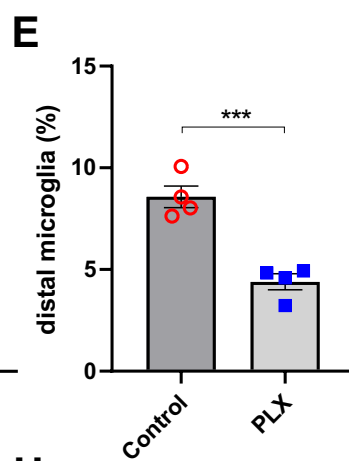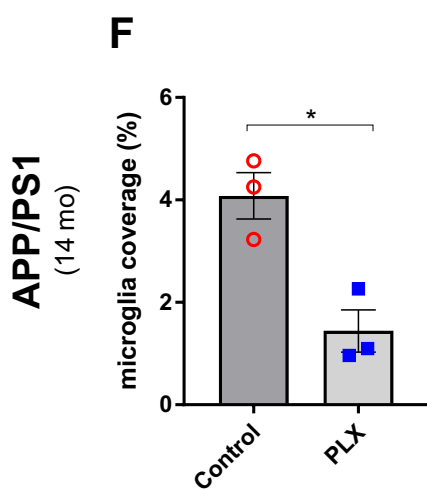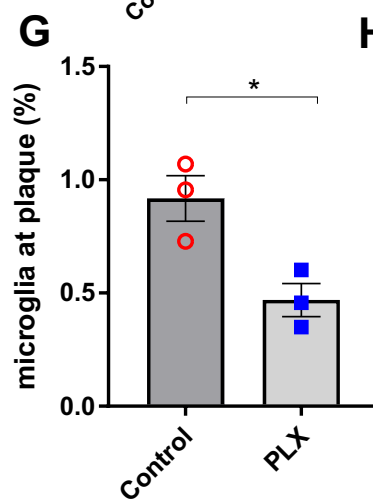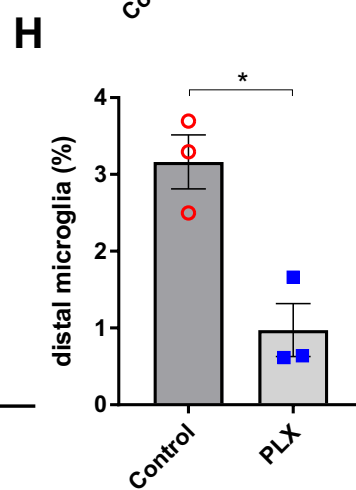

Supplement: Supplementary file 1 — Additional file 1: Figure S1. 2 weeks of PLX5622 administration partially eliminates microglia in 3xTg and APP/PS1 mice. Representative confocal immunofluorescent 10× images showing plaques (6E10, red) and microglia (Iba1, magenta) in the cortex and hippocampus in control versus PLX groups in 3xTg (A) and APP/PS1 mice (B). Scale bar represents 200 µm. Quantification shows an approximately 50% and 65% decrease in total microglia coverage with PLX treatment in 3xTg and APP/PS1 mice, respectively (C, F). PLX depletes plaque-associated microglia by around 40% in 3xTg and 50% in APP/PS1 (D, G) as compared to non-plaque associated microglia which are depleted around 50% (3xTg, E) and 70% (APP/PS1, H). Student’s t-test, *p < 0.05, ***p < 0.001. Data are presented as mean ± SEM (n = 3–4). [file 12974_2022_2532_MOESM1_ESM.pdf]

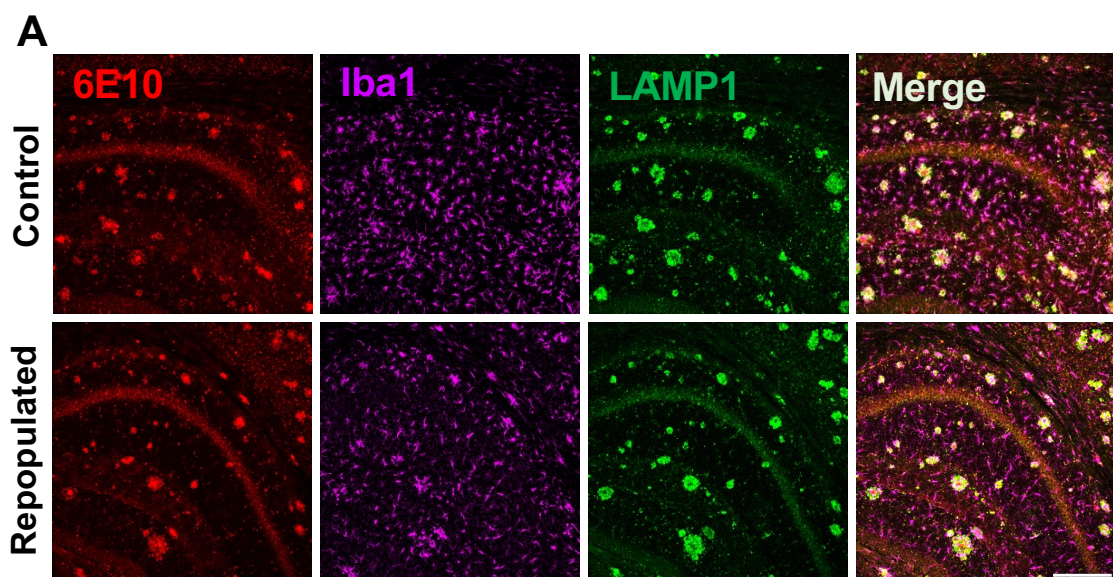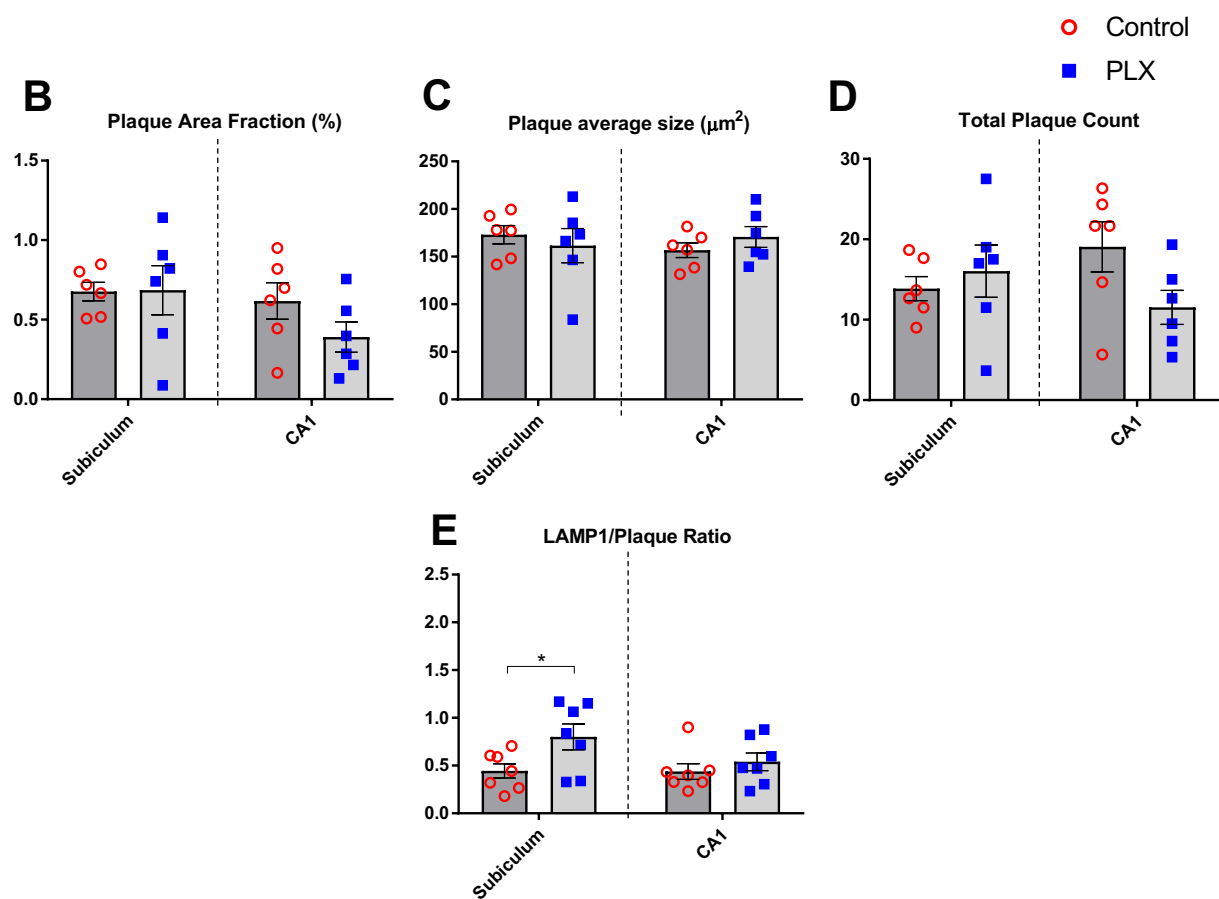

Supplement: Supplementary file 2 — Additional file 2: Figure S2. Repopulation of microglia does not ameliorate Aβ pathology and neuritic damage in APP/PS1 female mice. Representative immunofluorescent 20× images of the subiculum in control versus PLX-repopulated group, showing Aβ plaque (6E10, red), microglia (Iba1, magenta), and neuritic damage (LAMP1, green) (A). There was no difference in the total area (B), size (C) and number (D) of plaques between the control and PLX-treated groups. The ratio of plaque-associated neuritic damage to plaque load was similar between the control and PLX-repopulated group in CA1 but was significantly increased in the subiculum (E). Student’s t-test, *p < 0.05. Data are presented as mean ± SEM (n = 6). [file 12974_2022_2532_MOESM2_ESM.pdf]

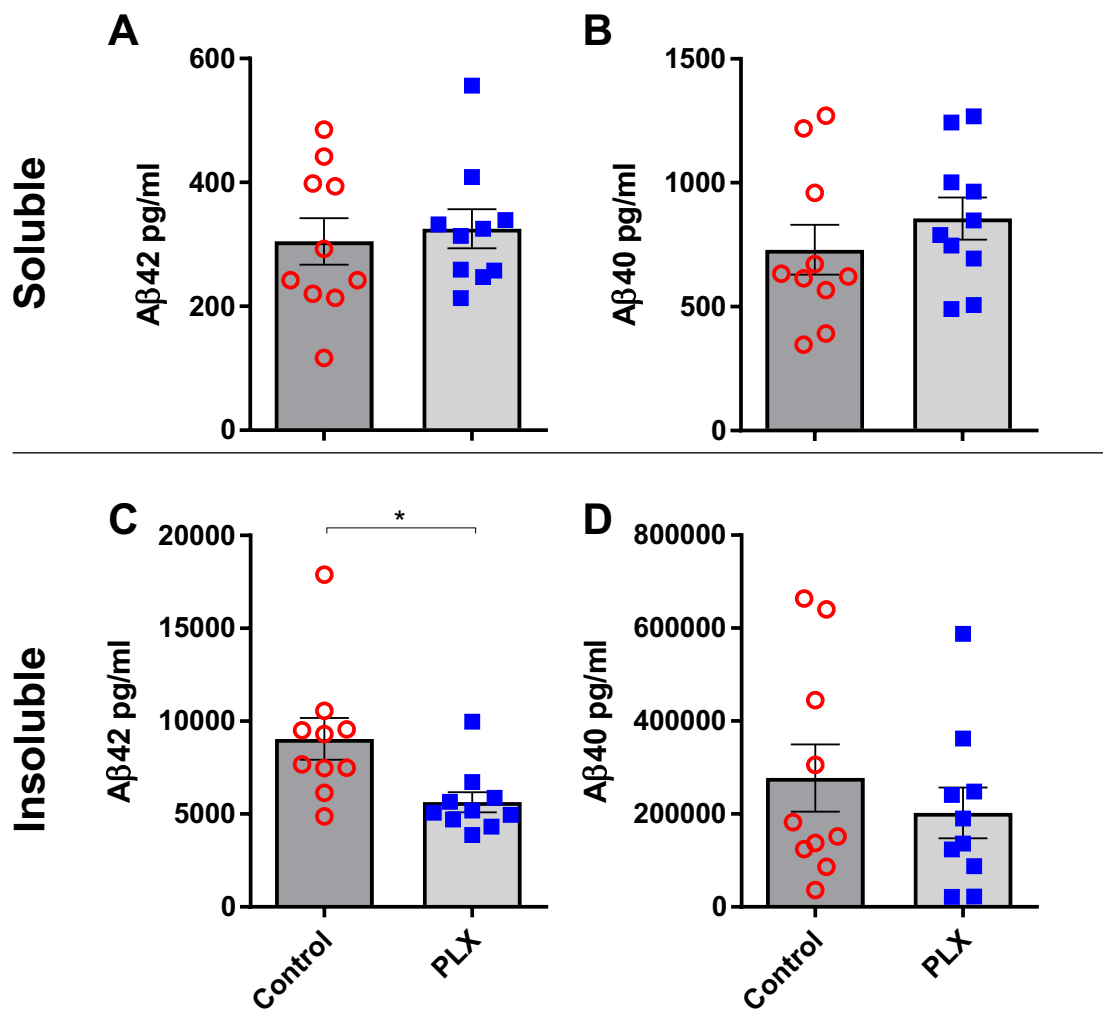

Supplement: Supplementary file 3 — Additional file 3: Figure S3. Repopulation of microglia causes subtle changes in Aβ peptide levels. Hippocampal lysates from 3xTg Cohort 1 were processed to separate soluble and insoluble fractions, which were used as substrates for ELISA for Aβ42 (A, C) or Aβ40 (B, D). Student’s t-test, *p < 0.05. Data are presented as mean ± SEM. n = 8–10. [file 12974_2022_2532_MOESM3_ESM.pdf]

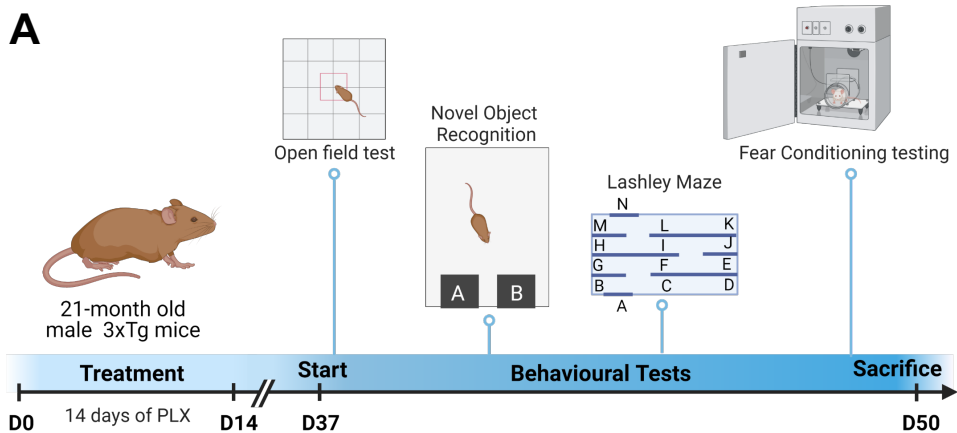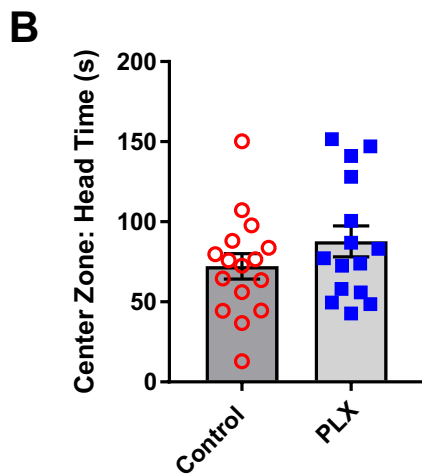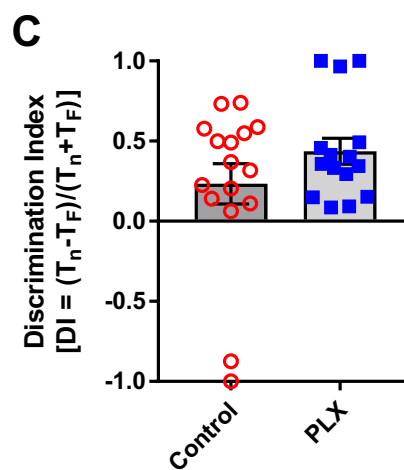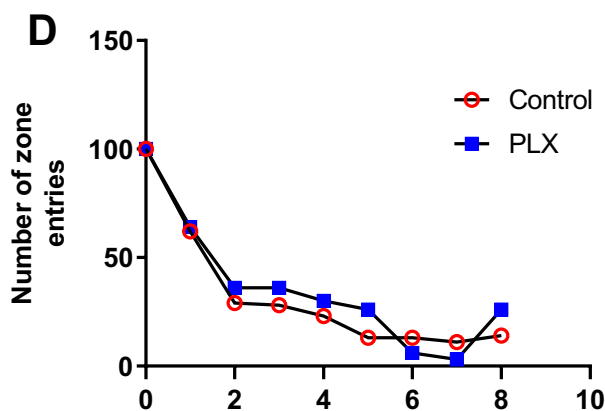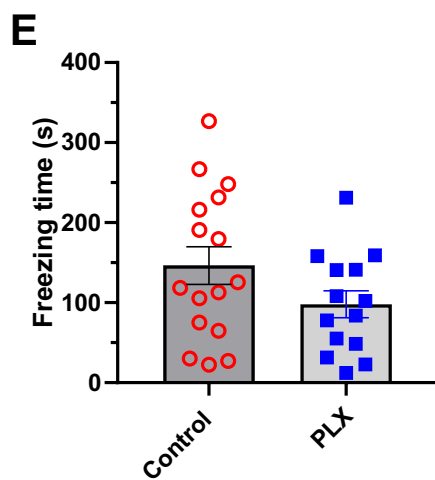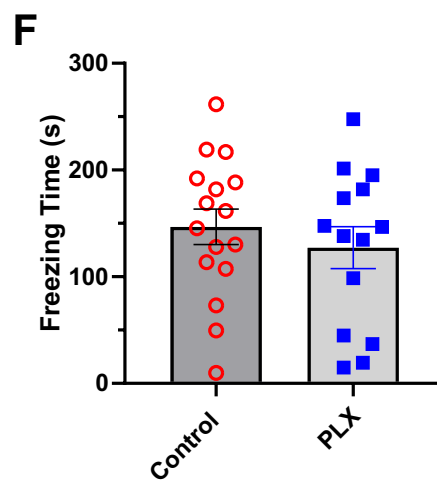

Supplement: Supplementary file 4 — Additional file 4: Figure S4. Repopulation of microglia does not alter behavior in 21-month-old male 3xTg mice. 21-month-old 3xTg mice were treated with control or PLX5622 chow for 14 days and then returned to control chow for the remainder of the experiment. After 22 days on control chow, behavioral assessments were conducted using Open Field, Novel Object Recognition, Lashley III maze, and Fear Conditioning (A). No differences were measured across groups in time spent in the open arena of the open field test (B) or the recognition index in the Novel Object Recognition task (C). To assess spatial memory in a stress-free environment the Lashley Maze was used as depicted. Mice were trained over 8 days to reach the pseudo-home cage from the start box in 10-min sessions each day. No change in the number of zone entries was observed between groups (D). No changes between the groups in the time spent freezing to the conditioned context (E) or the tone stimulus (F) were noted. Student’s t-test. Data are presented as mean ± SEM, n = 14–16. [file 12974_2022_2532_MOESM4_ESM.pdf]

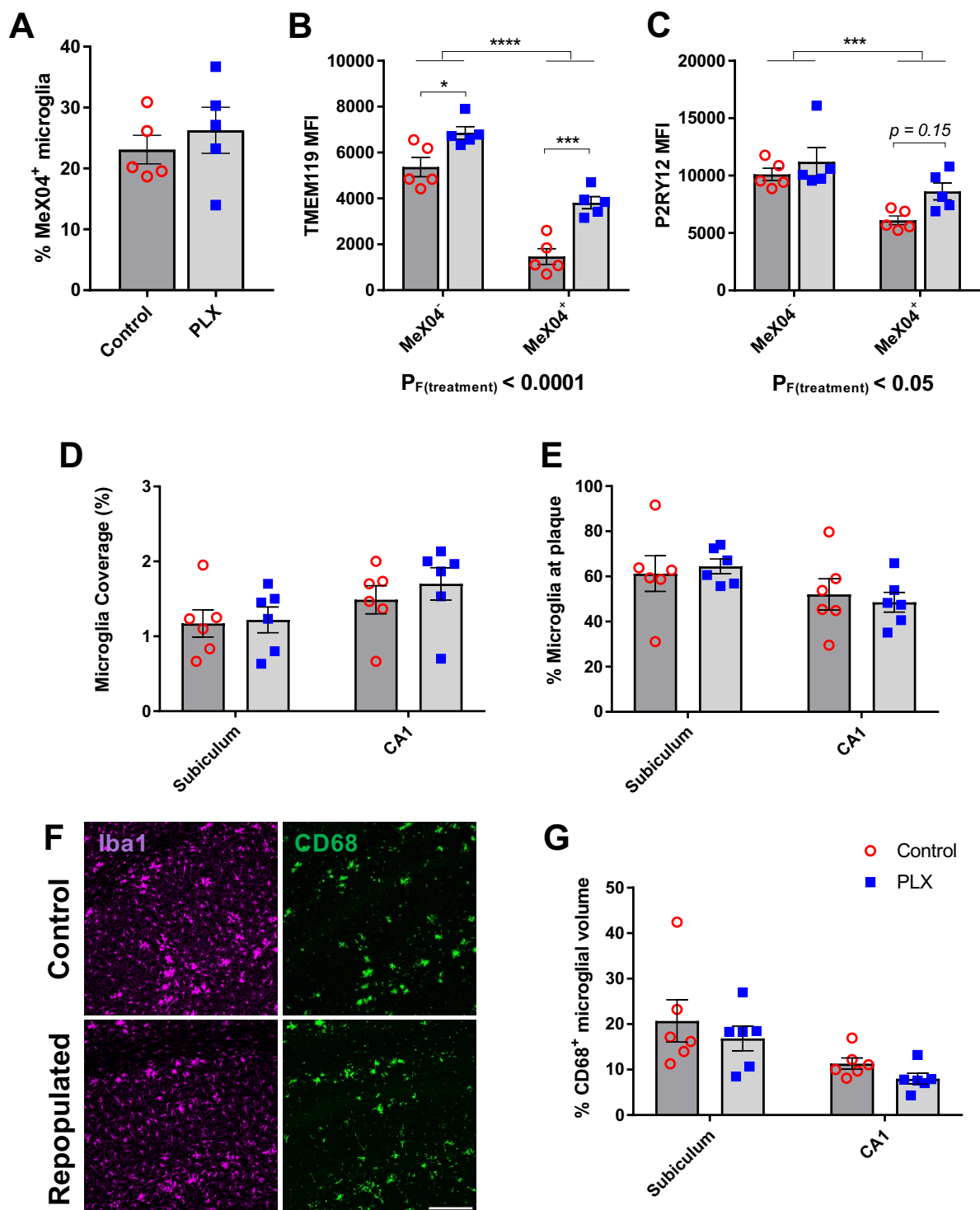

Supplement: Supplementary file 6 — Additional file 6: Figure S6. Repopulated microglia in male APP/PS1 show similar recruitment to plaques but different homeostatic markers levels. Flow cytometry revealed similar internalization of MeX04 between control and PLX-treated groups (A). TMEM119 levels were higher in MeX04− microglia in both conditions but was elevated by PLX treatment in both MeX04+ and MeX04− microglia (B). While PLX treatment increased P2RY12 levels, the effect was most apparent in MeX04+ microglia, which had lower P2RY12 levels in the control group (C). There were no differences in total microglial coverage (D) as well as microglial recruitment to plaque (E) between control and PLX treatment. Representative immunofluorescent 20× images of the subiculum in control and PLX-treated groups showing microglia (Iba1, magenta) and CD68 (green) (F). Scale bar represents 200 µm. Microglial CD68 levels did not change in both subiculum and CA1 regions with PLX treatment (G). Student’s t-test (A) and Two-way ANOVA with Tukey’s post hoc tests (B–E, G). Data are presented as mean ± SEM (n = 6). [file 12974_2022_2532_MOESM6_ESM.pdf]

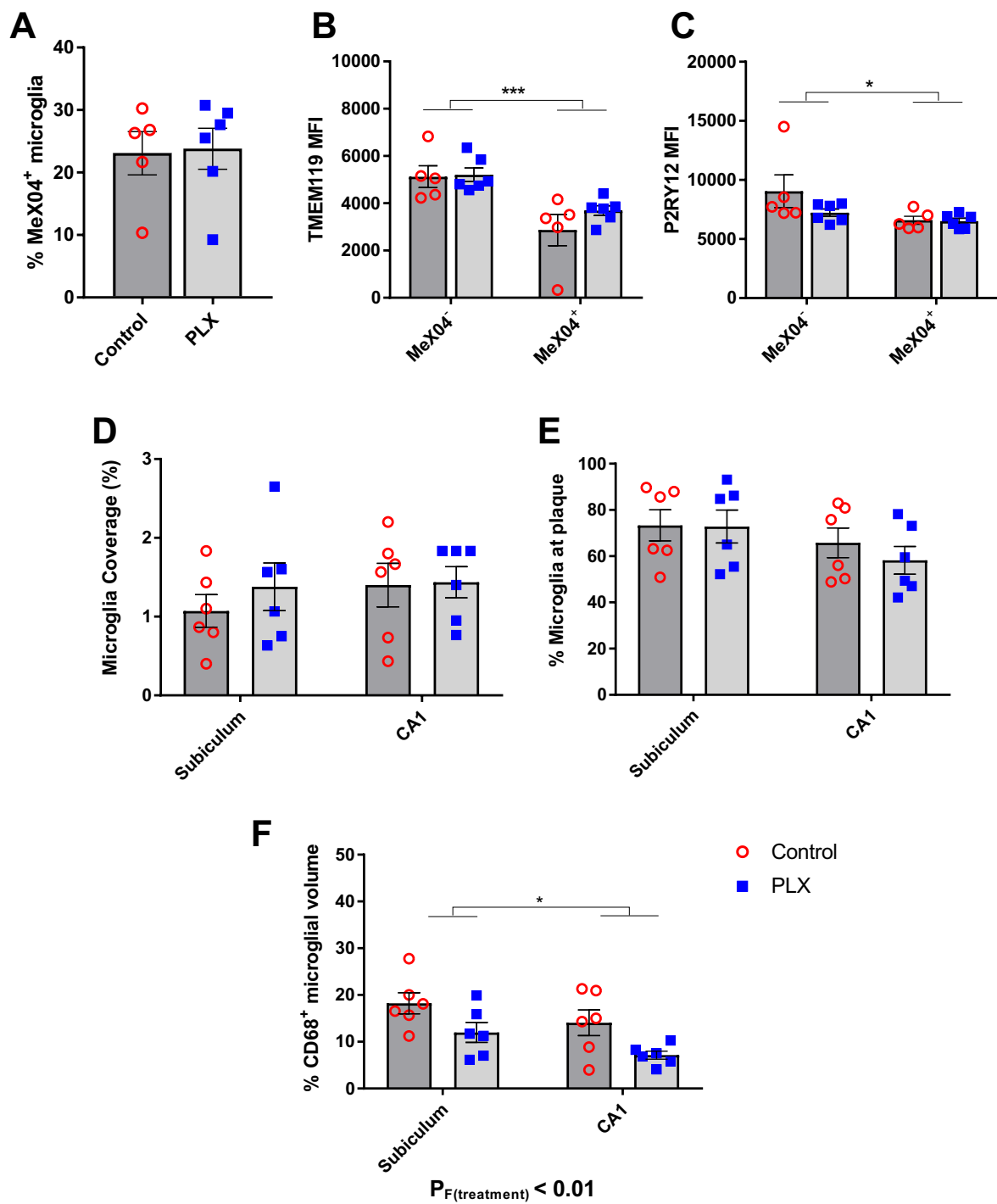

Supplement: Supplementary file 7 — Additional file 7: Figure S7. Repopulated microglia in female APP/PS1 show similar recruitment to plaques but different activated marker levels. Flow cytometry revealed similar internalization of MeX04 between control and PLX treatment groups (A). While both TMEM119 and P2RY12 levels were found to be lower in MeX04+ microglial, they did not change with PLX treatment (B, C). There was no difference in microglial total coverage (D) as well as their recruitment to plaque between treatments (E). Levels of microglial CD68 decreased in both subiculum and CA1 regions with PLX treatment (F). Student’s t-test (A) and Two-way ANOVA with Tukey’s post hoc tests (B–F). Data are presented as mean ± SEM (n = 6). [file 12974_2022_2532_MOESM7_ESM.pdf]

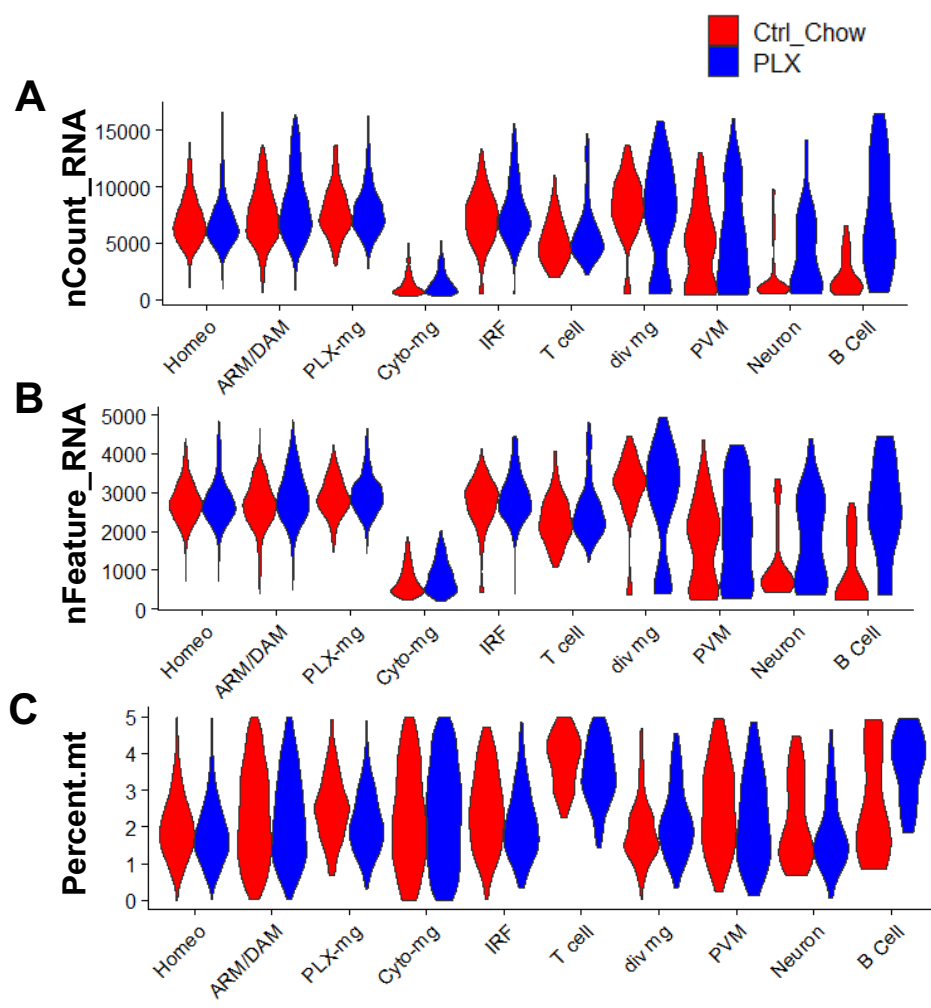

Supplement: Supplementary file 8 — Additional file 8: Figure S8. Quality Control metrics from scRNAseq dataset. Violin plots depicting the total number of detected RNA molecules (A), number of unique genes/features (B), and percentage of mitochondrial gene contamination (C) per cell post-quality control thresholding as described in “Methods” section. [file 12974_2022_2532_MOESM8_ESM.pdf]

**A**

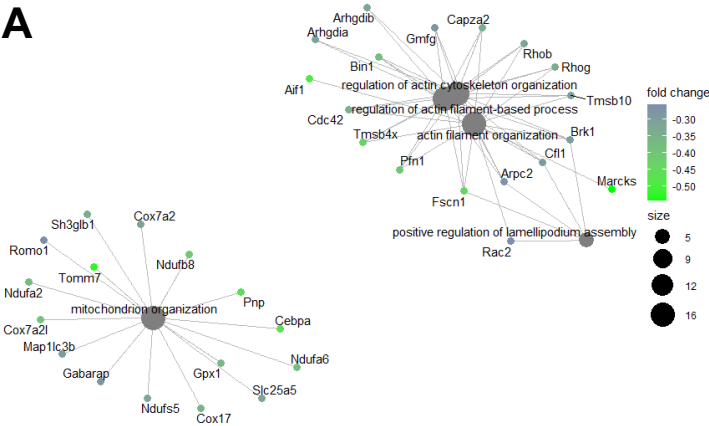

**B**

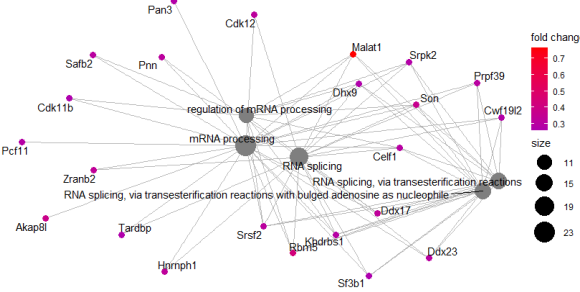

Supplement: Supplementary file 9 — Additional file 9: Figure S9. Gene Ontology Analysis of “Cyto-mg” cluster. Overrepresentation test (hypergeometric test) of genes detected to be significantly (padj < 0.05) up- (A) or down-regulated (B) by Seurat’s FindMarkers() function performed through clusterProfiler package. [file 12974_2022_2532_MOESM9_ESM.pdf]

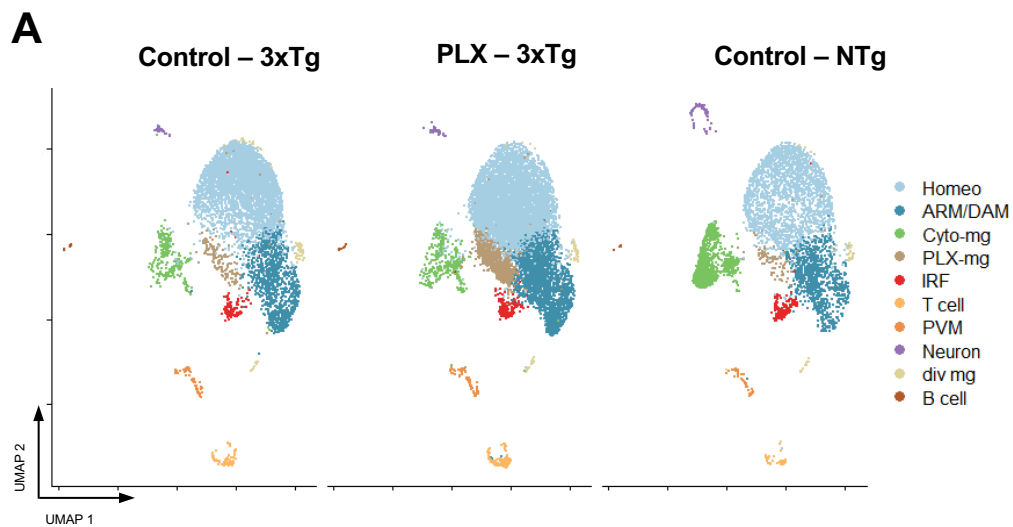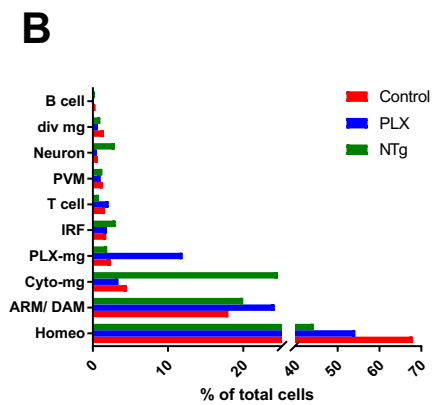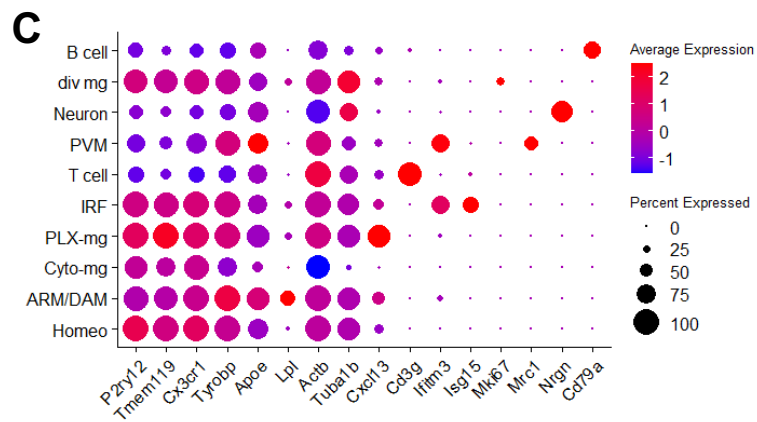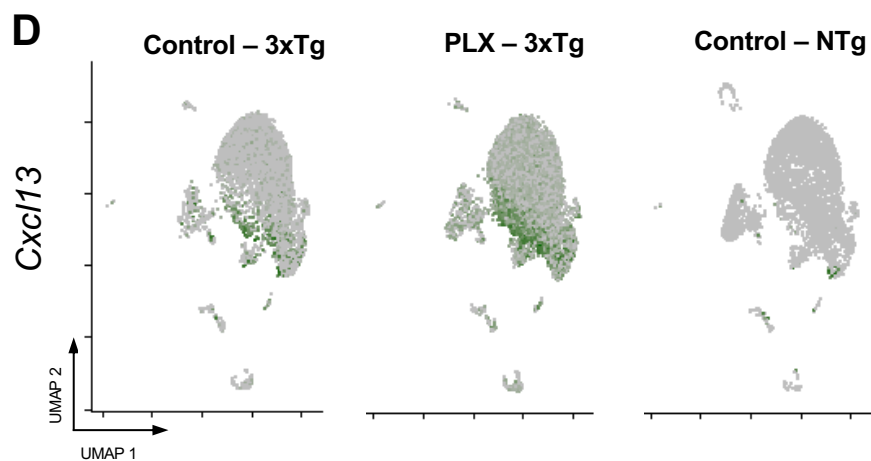

Supplement: Supplementary file 10 — Additional file 10: Figure S10. Comparisons of AD control and repopulated microglial gene expression with a non-AD control. The same dataset as shown in Fig. 4 is illustrated in relation to NTg, control-treated mice on UMAP space (A). All experimental groups were prepared in the same way and on the same day within < 3 h. However, since the 3xTg and NTg lines were separately maintained as homozygous lines for over a decade, Seurat’s anchoring algorithm was used to compare the data. Bar plot showing the proportions of CD45int/+ cells sequenced per cluster (B). Dot plot showing the expression of several important genes used in annotating the clusters (C). Comparison of Cxcl13 expression between control and PLX-treated 3xTg microglia and control-treated NTg microglia (D). Note the very few detected Cxcl13 transcripts in NTg microglia compared to 3xTg groups. [file 12974_2022_2532_MOESM10_ESM.pdf]
