# Supplementary material for: Repopulated microglia induce expression of Cxcl13 with differential changes in Tau phosphorylation but do not impact amyloid pathology
Source: J Neuroinflammation. 2022 Jul 4;19:173. doi: 10.1186/s12974-022-02532-9 (PMC9252071; doi:10.1186/s12974-022-02532-9)

3xTg Cohort 2

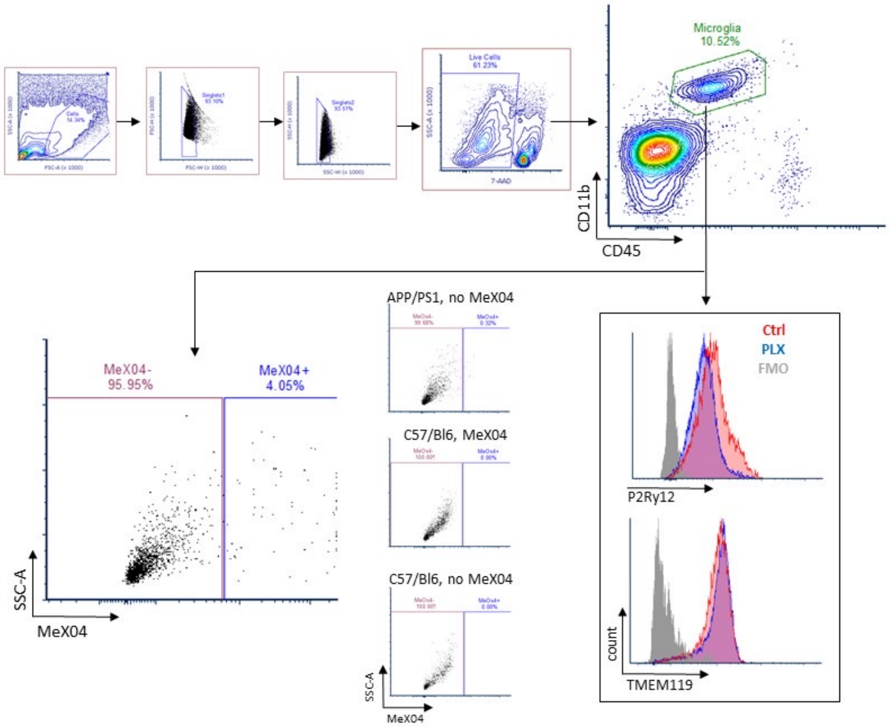

Supplement: Supplementary file 5 — Additional file 5: Figure S5. Gating strategy for 3xTg and APP/PS1 flow cytometry experiments. Briefly, debris was excluded based on SSC and FSC properties, and live cells were selected by gating for singlets and 7-AAD− events. Microglia were defined as CD45int CD11b+. MeX04 positive and negative events were determined with reference to APP/PS1, or non-transgenic C57BL/6 controls injected with MeX04 or vehicle (i.e., FMOs and negative controls). A representative sample from the second cohort of 3xTg mice is shown here. All flow cytometry experiments were gated as described above. [file 12974_2022_2532_MOESM5_ESM.pdf]
